# Physical activity and pain in people with cancer: a systematic review and meta-analysis

**DOI:** 10.1007/s00520-024-08343-3

**Published:** 2024-02-06

**Authors:** Mitchell Peters, Grace Butson, David Mizrahi, Linda Denehy, Brigid M. Lynch, Christopher T. V. Swain

**Affiliations:** 1 Cancer Science Unit, Faculty of Medicine, Dentistry and Health Sciences, University of Melbourne, Melbourne, Australia; 2 Princess Alexandra Hospital, Woolloongabba, QLD Australia; 3 Peter MacCallum Cancer Centre, Melbourne, VIC Australia; 4 The Daffodil Centre, The University of Sydney, a Joint Venture With Cancer Council NSW, Sydney, NSW Australia; 5 Department of Physiotherapy, Faculty of Medicine, Dentistry and Health Sciences, Melbourne School of Health Sciences, University of Melbourne, Level 7, Alan Gilbert Building, 161 Barry St, Melbourne, VIC 3010 Australia; 6 Cancer Epidemiology Division, Cancer Council Victoria, Melbourne, Australia; 7 Centre for Epidemiology and Biostatistics, Melbourne School of Population and Global Health, University of Melbourne, Melbourne, Australia; 8 Physical Activity Laboratory, Baker Heart and Diabetes Institute, Melbourne, Australia

**Keywords:** Cancer pain, Exercise, Neoplasm, Review

## Abstract

**Purpose:**

Physical activity can provide analgesic benefit but its effect on cancer-related pain is unclear. This review synthesised and appraised the evidence for the effect of physical activity on pain in people living with or beyond cancer.

**Methods:**

A systematic search of Ovid Medline and Embase was performed to identify randomised controlled trials (RCTs), randomised cross-over studies (RXTs), and prospective observational studies that examined physical activity and pain outcomes in adults living with or beyond cancer. Meta-analyses were performed to generate effect estimates. Risk of bias was assessed, and the GRADE system was used to assess evidence quality.

**Results:**

One hundred twenty-one studies (*n* = 13,806), including 102 RCTs, 6 RXTs, and 13 observational studies, met the criteria for inclusion. Meta-analyses of RCTs identified a decrease in pain intensity (*n* = 3734; standardised mean difference (SMD) − 0.30; 95% confidence interval (CI) − 0.45, − 0.15) and bodily pain (*n* = 1170; SMD 0.28; 95% CI 0.01, 0.56) but not pain interference (*n* = 207; SMD − 0.13, 95% CI − 0.42, 0.15) following physical activity interventions. Individual studies also identified a reduction in pain sensitivity but not analgesic use, although meta-analysis was not possible for these outcomes. High heterogeneity between studies, low certainty in some effect estimates, and possible publication bias meant that evidence quality was graded as very low to low.

**Conclusion:**

Physical activity may decrease pain in people living with and beyond cancer; however, high heterogeneity limits the ability to generalise this finding to all people with cancer or to specific types of cancer-related pain.

**Supplementary Information:**

The online version contains supplementary material available at 10.1007/s00520-024-08343-3.

## Introduction

Cancer-related pain includes pain caused by a cancer or its treatments related and is one of the most common symptoms reported by people living with or beyond cancer. Pain is experienced by approximately 60% of all people undergoing treatment for cancer and continues to be reported by 40% of patients after completing their treatment [147, 148]. More than one in four people with cancer describe their pain as severe, and the impact of cancer-related pain can affect every aspect of a person’s life and can be a reason for cessation of treatment [90, 102, 147, 148].

Successful management of cancer-related pain is complex, often reliant on pharmacological intervention, and limitations in the scientific evidence for treatment possibilities compound existing patient, provider, and system barriers [43, 59]. An improved understanding of non-pharmacological treatment strategies for pain was a primary goal identified in the 2019 Australian Strategic Action Plan for Pain Management and has been highlighted by the American Centre for Disease Control as an essential step in improving pain management [44, 54]. This sentiment has been echoed in qualitative studies, which indicate that people with cancer would like more treatment options for pain, including non-pharmacological strategies [90]. Unfortunately, many do not always feel they are supported when seeking non-pharmacological pain treatments [90].

Physical activity, including structured exercise and incidental or leisure time activities, can provide analgesic benefit and is considered an important component of pain management for non-cancer pain. In healthy populations, exercise increases the threshold for experimentally induced pain and prevents the development of several chronic pain conditions [64, 85, 134]. Benefits can be seen after just one session of exercise and have been attributed to the effect of exercise on several central, immune system, and psychological pathways [85, 123]. In several clinical populations (e.g. back pain, arthritic conditions, fibromyalgia), exercise can promote analgesia and reduce pain-related disability [134]. However, an analgesic effect is not evident in all patient populations who experience pain, with some populations (e.g. chronic fatigue syndrome, chronic neck pain) experiencing exacerbations of pain with exercise in some studies [85, 123]. This can present a major barrier to engagement and adherence in physical activity programmes, potentially leading to long-term sedentary behaviours and poor health outcomes.

Although there is some evidence that supports a decrease in cancer-related pain following exercise [79], collectively, the evidence remains sparse. No studies examining the effects of physical activity or exercise on pain sensitivity in people who have cancer-related pain were identified in two recent reviews [14, 123]. Further, recent guidelines addressing the effects of exercise on health outcomes in people with cancer, which were developed by a group of international exercise oncology experts, state that the evidence is insufficient to support prescription of exercise for cancer-related pain [27, 131]. Several international cancer pain management guidelines do not include exercise as a possible treatment [89]. Similarly, many cancer-related pain management documents developed for consumers provide limited information on the potential benefit of physical activity or exercise on cancer pain [28]. A greater understanding of the effects of exercise on cancer-related pain is warranted. This review will examine the evidence for an effect of physical activity on pain outcomes in people with cancer.

## Methods

This systematic review and meta-analysis were conducted in accordance with the PRISMA 2020 Statement [108]. The review protocol was uploaded to PROSPERO prior to commencement (CRD42021267826).

### *Searchstrategy*

A systematic search of Ovid Medline and Embase electronic databases was initially performed on the 26th of August 2021. This search was updated on 5th of December 2023. The search strategy included a combination of Medical Subject Headings (MESH) and free text terms, and an example search is presented in Supplementary Table 1. Reference lists from other reviews were manually searched to identify any references that were not otherwise found. No date limits were applied to the search or eligibility criteria.

### Eligibility criteria

Eligible studies included peer-reviewed parallel group randomised controlled trials (RCTs), randomised cross-over trials (RXTs), and prospective observational studies. Participants could include adults (> 18 years) who have cancer or survivors of adult cancer. Physical activity could include structured exercise (e.g. moderate intensity continuous exercise) or less structured physical activity (e.g. leisure time physical activity). Comparisons such as usual care, activity quantity (e.g. inactivity or low activity), or activity type (e.g. resistance exercise compared to aerobic exercise) were included. Eligible outcomes included pain prevalence, intensity, impact, quality, sensitivity, or analgesic use. Studies were excluded if they included non-randomised interventions or cross-sectional analyses, participants with childhood cancer or survivors of childhood cancer, and interventions or exposures such as passive movement and manual therapy, highly specific therapy (e.g. jaw strengthening, swallowing exercises), or physical activity combined with a second intervention or exposure (e.g. diet, relaxation therapy). Studies with no comparison condition (e.g. single arm trials) were also excluded. Pain that was clearly not related to cancer (e.g. mechanical low back pain) or broader outcome measures (e.g. ‘cancer symptoms’) were excluded. Only studies published in English were included.

### Screening, extraction, and appraisal

Following duplicate removal, all eligible title and abstracts returned via the search were screened independently by two reviewers (MP, CS, DM, or BL). Studies that were clearly not relevant were excluded. Two reviewers (MP, CS) then screened the full texts of all remaining references to determine the final eligibility for inclusion with disagreement resolved via discussion. One investigator performed data extraction and the risk of bias assessment for each study (MP, GB, or DM) using a pre-piloted data extraction form, which was then reviewed by the senior investigator (CS). Extracted data included study details (author, year), population (e.g. cancer type, stage, participant, sample size), intervention or exposure details (e.g. physical activity type) and assessment method (e.g. self-report), comparison or control condition, outcome definitions, confounding factors used for adjustment, and outcome values. Web plot digitiser was used to extract outcome data presented visually [42]. Risk of bias was performed using the Cochrane Collaboration Tool for randomised controlled trials and the Risk of Bias in Studies of Exposures (ROBINS-E) tool for prospective observational studies [73, 100]. The overall quality of evidence for a physical activity—cancer pain relationship—was appraised using the Grading of Recommendations Assessment, Development, and Evaluation (GRADE) system [66].

### Meta-analysis

For all extracted outcomes, data were summarised and presented descriptively. Where study design, exposures, outcomes, and analyses were defined consistently in at least three separate studies, random-effects meta-analysis of final study values was used to generate a standardised mean difference (SMD) with 95% confidence interval (CI). Studies with multiple intervention arms (e.g. two types of exercise) were combined into a single group, as per Cochrane recommendations [74]. Statistical heterogeneity was quantified using the *I*^2^ statistic. Where there was more than moderate heterogeneity (*I*^2^ > 40%), subgroup analyses were performed to examine the influence of cancer type or physical activity type and setting. Publication bias was assessed by visual inspection of funnel plots. All meta-analyses were performed using Stata version 16 (Stata Corporation, College Station, TX, USA).

## Results

### Search results

**Fig. 1:**
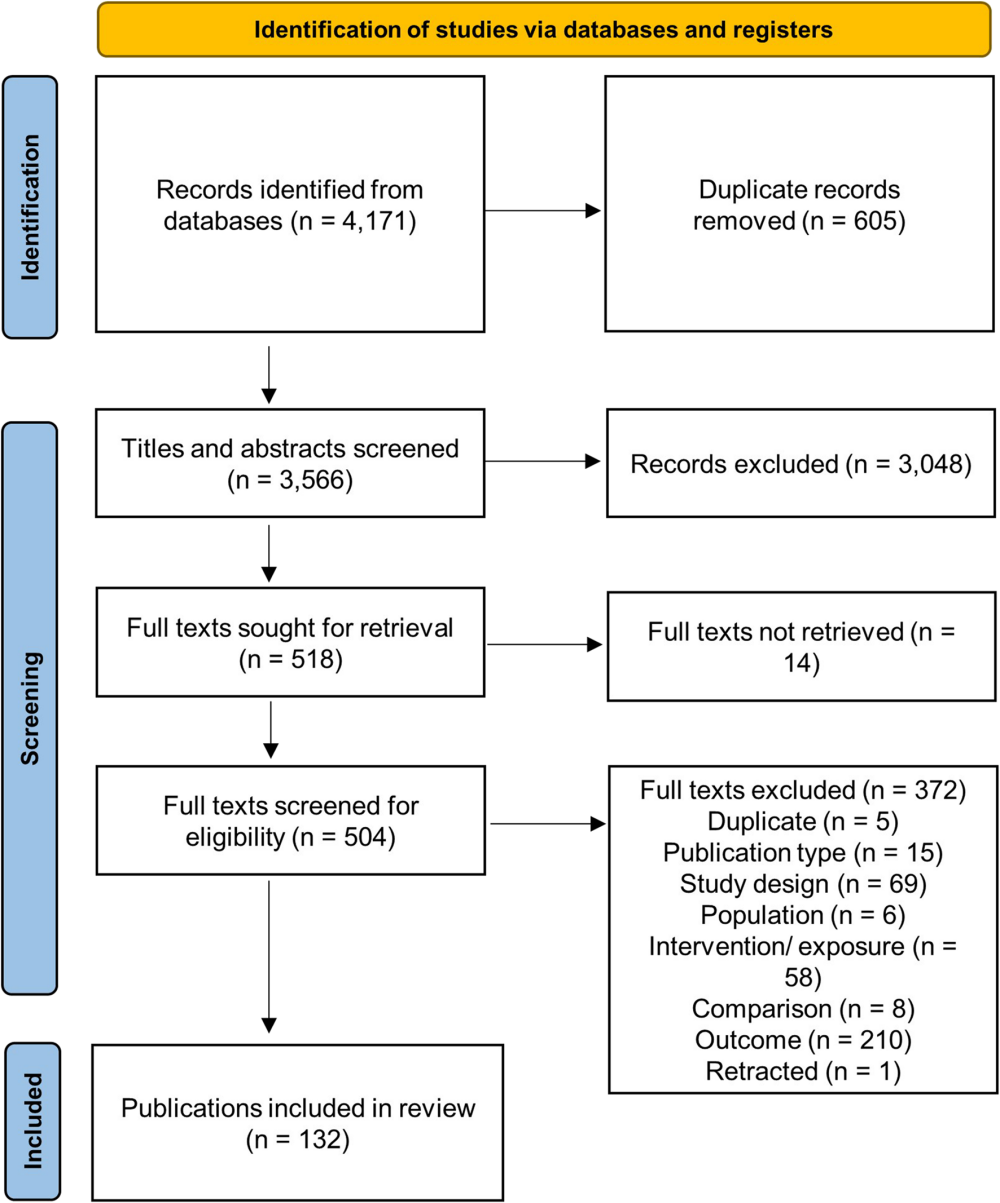
PRISMA chart

Search results are presented in Fig. 1. From 4171 records first identified, 3566 titles and abstracts and 504 full texts screened, there were 132 publications from 121 studies included in the final review. Studies included 102 RCTs [1, 2, 4, 6–12, 15, 16, 18, 19, 21–24, 26, 29–32, 35, 37, 38, 40, 41, 45–52, 55, 57, 61–63, 65, 67–72, 75–81, 83, 84, 86–88, 91–99, 101, 103–106, 109, 110, 112–114, 116, 118–122, 124–127, 129, 130, 132, 133, 136–140, 143, 145, 146, 149–151, 153–157], six randomised RXTs [17, 34, 36, 39, 142, 152], and 13 observational studies [3, 13, 20, 33, 53, 56, 58, 60, 82, 115, 128, 135, 144, 158]. Overall, these studies included 13,806 participants.

### Study characteristics

Study characteristics are presented in Supplementary Table 2–4. Median sample sizes were 60 (range 10 to 577) in RCTs, 19 (range 10 to 21) in RXTs, and 199 (range 42 to 1937) in prospective observational studies. Participants included patients undergoing treatment and survivors of breast (RCT = 48, RXT = 3, observational = 7), prostate (RCT = 10, observational = 1), lung (RCT = 7, observational = 2), colorectal (RCT = 5), head and neck (RCT = 2, observational = 2), haematological (RCT = 2), bladder (RCT = 1), gastrointestinal (RCT = 6), ovarian (RCT = 1), or combined/ non-specific sites (RCT = 15, RXT = 3, observational = 3). Interventions included general physical activity (RCT = 20), aerobic exercise (RCT = 21, RXT = 2), resistance exercise (RCT = 16, RXT = 3), combined aerobic and resistance exercise (RCT = 39, RXT = 1), aquatic exercise (RCT = 4), yoga (RCT = 4), Pilates (RCT = 3), dance (RCT = 3), Tai chi (RCT = 1), or Qigong (RCT = 1, RXT = 1). In observation studies, physical activity was assessed via self-report (*n* = 9), accelerometry/ smart-phone app (*n* = 6), or was unclear (*n* = 1). The comparison condition included usual care or activity (RCT = 81, RXT = 1), type of physical activity or exercise (RCT = 14, RXT = 1), timing of physical activity (RCT = 3), training dose (RCT = 4, RXT = 4), and setting, delivery method, or promotion strategy (RCT = 6). Pain intensity was the most common outcome (RCT = 68, RXT = 5, observation = 10), followed by bodily pain (RCT = 33, RXT = 1, observation = 2), pain interference (RCT = 8, RXT = 1, observation = 1), neuropathic pain (RCT = 4, observational = 1), pain quality (RCT = 1), pain presence (RCT = 1, observation = 2), bone pain (RCT = 1), analgesic use (RCT = 4, observational = 1), pain sensitivity (RCT = 4, RXT = 1), and pain frequency (RCT = 1, observation = 1).

## Risk of bias

Risk of bias results are presented in Supplementary Table 5–7. All RCTs scored high for performance bias as it is not possible to blind participants from their exercise intervention status. Other reasons for bias in RCTs included unclear bias arising from randomisation procedures [31, 47–52, 55, 65, 71, 77, 80, 88, 98, 99, 105, 106, 113, 116, 122, 130, 138–140, 151, 156] and unclear or high bias for greater than 10% attrition or less than 90% intervention adherence [1, 2, 6–9, 11, 15–19, 21, 23, 24, 26, 29, 32, 40, 41, 49, 50, 55, 61, 67–71, 75, 76, 79, 80, 84, 86–88, 93, 94, 98, 103, 106, 109, 110, 112–114, 118, 119, 129, 133, 138, 143, 150, 151, 153, 154, 157]. In RXTs, one study was judged to have high bias arising from potential carry over effects [142], and one was judged to have high bias due to deviations from the intended intervention [152]. All observation studies had at least moderate risk of bias due to the likely presence of confounding, self-reported measurement of physical activity, or possible exposure departures (i.e. a change in physical activity). Seven studies had serious risk of bias as they did not adjust for minimally important confounders [33, 56, 58, 82, 128, 135, 144].

### Physical activity and pain intensity

**Fig. 2:**
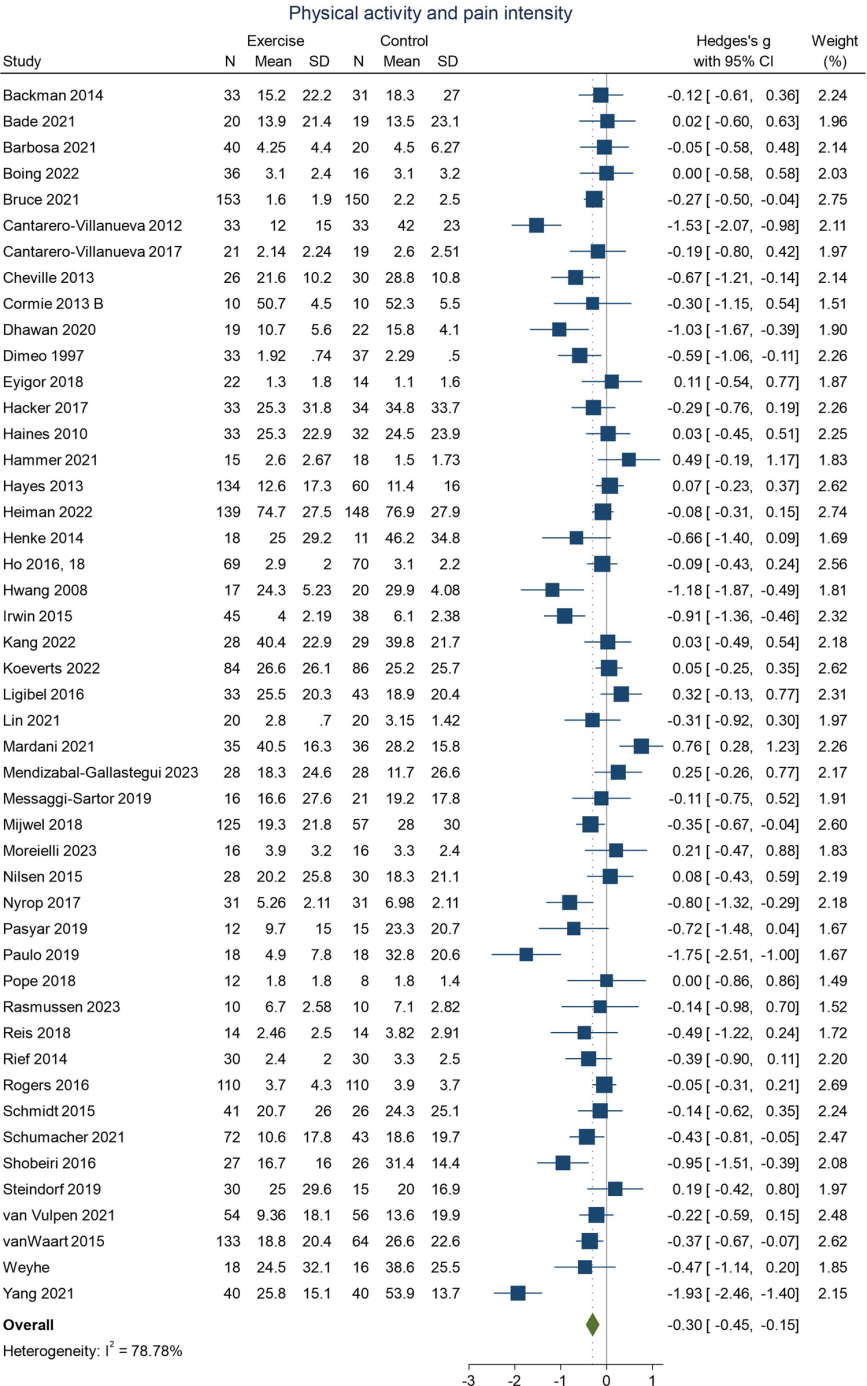
Physical activity and pain intensity in people with cancer. An effect size < 0 indicates a decrease in pain intensity or interference following physical activity intervention

Meta-analysis of RCTs that examined the effect of physical activity compared to a control condition on pain intensity is presented in Fig. 2. Overall, physical activity interventions resulted in a decrease in pain intensity (studies = 47; *n* = 3734; SMD − 0.30; 95% CI − 0.45, − 0.15; *I*^2^ = 79%). There was high heterogeneity and evidence of possible publication bias in the funnel plots (Supplementary Fig. 7).

Subgroup analyses (Supplementary Figs. 1–3) demonstrated decreases in pain following physical activity interventions for breast cancer (studies = 24; *n* = 2340; SMD − 0.34; 95% CI − 0.54, − 0.13; *I*^2^ = 81%) and studies that examined other or multiple cancers (studies = 16; *n* = 1025; SMD − 0.35; 95% CI − 0.62, − 0.08; *I*^2^ = 78%) but not for lung (studies = 3; *n* = 105; SMD − 0.20; 95% CI − 0.58, 0.18; *I*^2^ = 0%) or prostate cancer (studies = 4; *n* = 264; SMD 0.04; 95% CI − 0.51, 0.59; *I*^2^ = 78%). Subgroup analyses also identified larger effect estimates for interventions that featured resistance training (studies = 9; *n* = 697; SMD − 0.35; 95% CI − 0.61, − 0.09; *I*^2^ = 57%) or combined modalities (studies = 17; *n* = 1507; SMD − 0.28; 95% CI − 0.50, − 0.05; *I*^2^ = 77%) than for physical activity interventions alone (studies = 6; *n* = 438; SMD − 0.11; 95% CI − 0.43, 0.21; *I*^2^ = 56%). Larger effects for supervised interventions (studies = 27; *n* = 1945; SMD − 0.35; 95% CI − 0.53, − 0.17; *I*^2^ = 70%) compared with unsupervised interventions (studies = 8; *n* = 603; SMD − 0.27; 95% CI − 0.61, − 0.08; *I*^2^ = 72%) were also evident. However, the heterogeneity between studies remained high following subgroup analyses for each cancer site, physical activity type, and supervised or unsupervised intervention.

Individual RCTs not included in the meta-analysis (Supplementary Table 8) had either null findings [50, 65, 78, 150] or small decreases in pain intensity for physical activity interventions compared to a usual care control [2, 5, 91, 109, 110, 114, 143]. One RXT did not identify a difference in pain following exercise or usual care [142]. Most prospective observational studies suggested an association between more physical activity and reduced pain symptoms [20, 58, 82, 115, 128, 135, 144]. Three observational studies found no definitive relationship [33, 56, 158].

### Physical activity and bodily pain

**Fig. 3:**
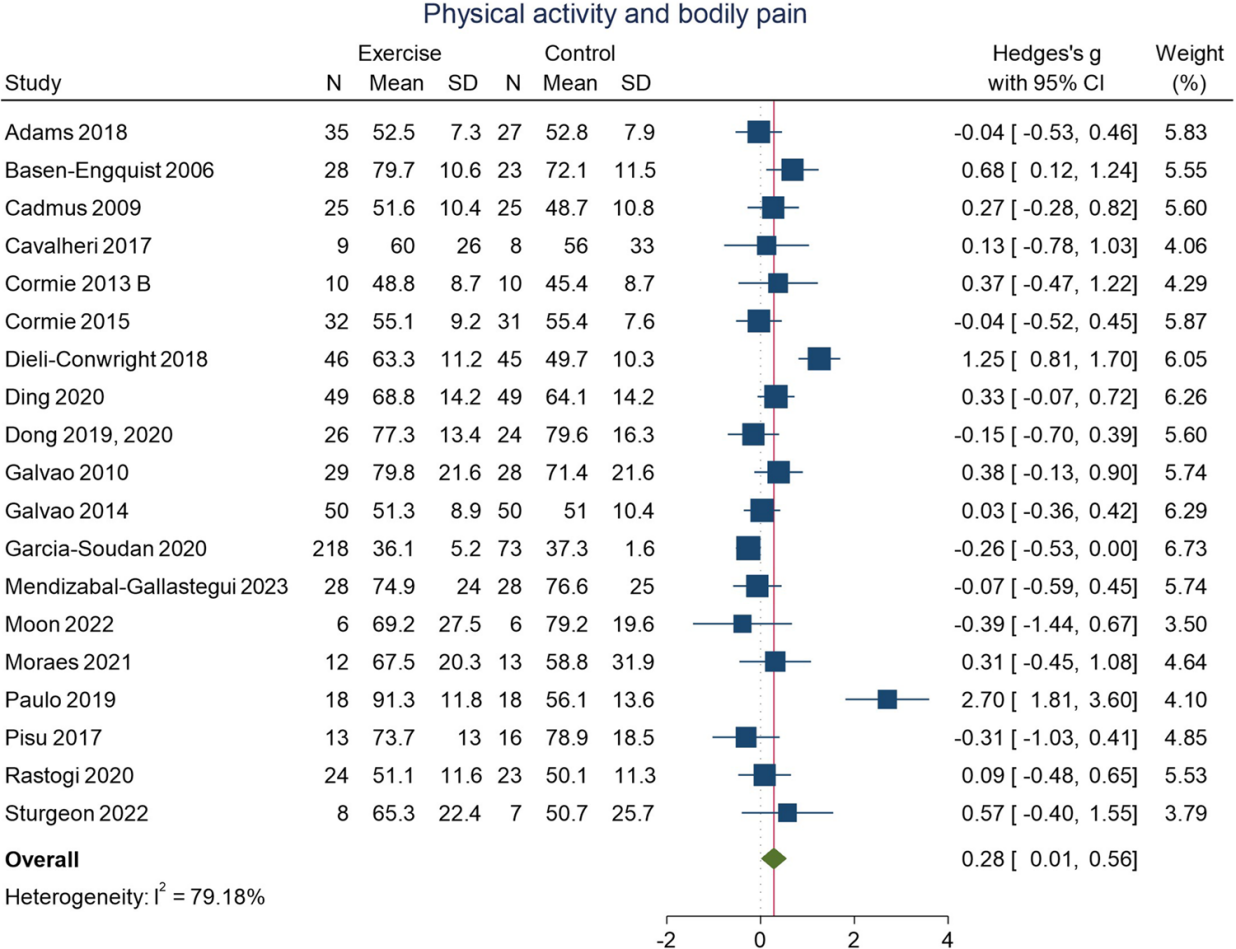
Physical activity and bodily pain in people with cancer. An effect size > 0 indicates an improvement in bodily pain following physical activity intervention

Meta-analysis of RCTs that examined the effect of physical activity on bodily pain is presented in Fig. 3. Overall, physical activity interventions resulted in a decrease (i.e. higher score) in bodily pain (i.e. less pain; studies = 19; *n* = 1170; SMD 0.28; 95% CI 0.01, 0.56; *I*^2^ = 79%). There was high heterogeneity and evidence of possible publication bias in the funnel plots (Supplementary Fig. 8).

Heterogeneity remained high following subgroup analyses (Supplementary Figs. 4–7) that stratified for cancer site, physical activity type, and intervention supervision status. Effect estimates may have been higher for breast cancer (studies = 8; *n* = 609; SMD 0.63; 95% CI 0.00, 1.26; *I*^2^ = 90%), for supervised interventions (studies = 10; *n* = 724; SMD 0.39; 95% CI − 0.14, 0.92; *I*^2^ = 92%), and for interventions with combined modalities (studies = 11; *n* = 823; SMD 0.33; 95% CI − 0.15, 0.81; *I*^2^ = 91%). However, wide confidence intervals as well as high heterogeneity limit the certainty of these findings.

Findings from RCTs, RXTs, and observational studies that examined changes in bodily pain following physical activity intervention compared to control that were not included in the meta-analyses had mostly null findings [3, 6, 23, 31, 34, 38, 136, 146, 157]. Some individual studies did suggest possible decrease in bodily pain following physical activity [13, 22, 119].

### Physical activity and pain interference

**Fig. 4:**
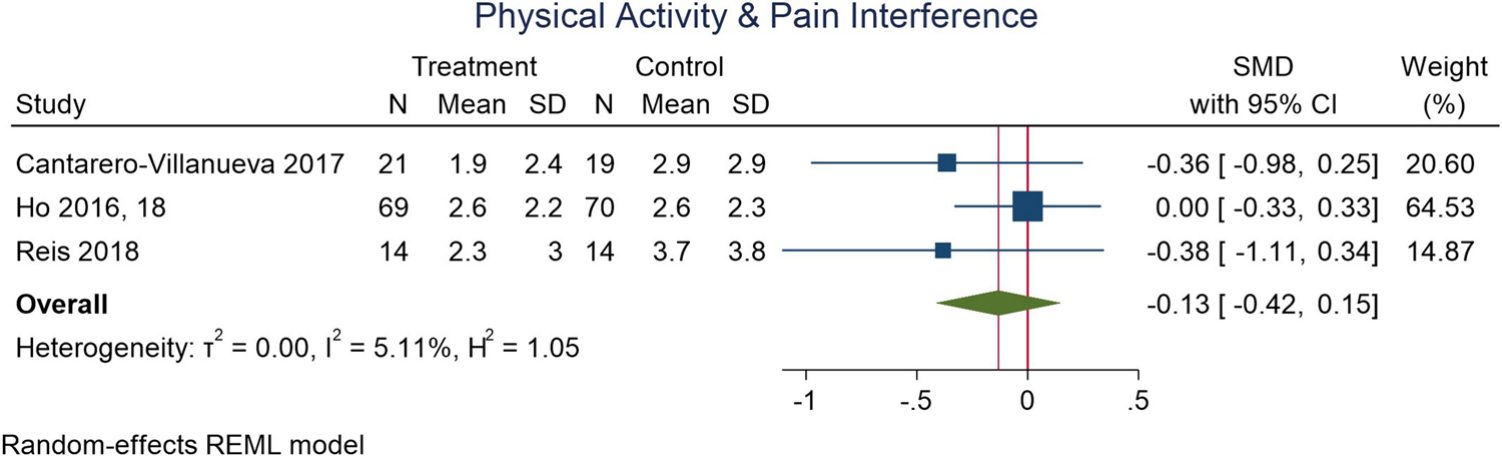
Physical activity and pain interference in people with cancer. Although an effect size < 0 suggests a decrease in pain interference after physical activity intervention, as the confidence intervals cross the null, the certainty of this finding is limited

Results for an effect of physical activity on pain interference were unclear. Meta-analysis (Fig. 4) noted no definitive change in interference following interventions (studies = 3; *n* = 207; SMD − 0.13; 95% CI − 0.42, 0.15; *I*^2^ = 5%). In studies not included in the meta-analysis (Supplementary Table 8), two RCTs identified no change [57, 78] and two identified small to moderate decreases in pain interference [38, 79]. In a RXT, there were small decreases in pain interference 24 and 72 days following resistance training [36].

### Physical activity and pain sensitivity

Five studies, including four parallel group RCTs and one RXT, examined the relationship between physical activity and pain sensitivity in people with cancer (Supplementary Table 8). Each study documented a decrease in pain sensitivity at some affected sites following physical activity. In women with breast cancer undergoing chemotherapy, high-intensity interval training combined with resistance or aerobic training decreased pain sensitivity when compared to usual care, with stronger effects with resistance training [96]. In survivors of any cancer, a single bout of low or high intensity exercise decreased pain sensitivity in exercising but not non exercising muscles, with greater effects for higher intensity exercise [34]. A 2-week period of low or high intensity exercise decreased post-exercise pain sensitivity at both sites, with exercise eliciting a medium effect in exercising muscles and a small effect in non-exercising muscles [34]. In breast cancer survivors, both aquatic therapy and resistance training reduced pain sensitivity at select sites [30, 120]. In colon cancer survivors, lumbopelvic exercises and light aerobic exercise decreased pain sensitivity at lumbar and some, but not all abdominal sites, and not at the second metacarpal [29].

### Physical activity and analgesic use

Four RCTs reported on the use of pain medication, with no suggestion that physical activity interventions decrease analgesic use more than control conditions [11, 38, 79, 138]. One RCT reported that analgesic use in people with lung cancer scheduled to receive surgery was higher in the exercise intervention group compared to the control group after 3 months of activity [138].

### Physical activity type and pain outcomes

Twelve RCTs and one RXT compared types of exercise (Supplementary Table 8). Results of individual studies cannot support any optimal type of exercise. Some studies showed no difference in effect for type [80, 88, 106, 152, 153, 156], while others showed larger effects of aerobic exercise compared to resistance [40, 63, 129], or for resistance and high intensity interval training compared to aerobic exercise combined with high intensity interval training [19, 113]. In other studies, aquatic exercises may have been more effective than land exercises [4], and Pilates more effective than circuit exercises [11].

### Physical activity dose and pain outcomes

Five RCTs, four RXTs, and three observational studies examined the relationship between intervention or physical activity dose and pain outcomes (Supplementary Table 8—9). Results either favoured a higher dosage (i.e. a higher dose led to a greater decrease in pain) [16, 22, 34, 40, 58], a low-moderate dosage/ intensity [8], or did not identify differences by dose [36, 38, 39, 104].

### Timing of physical activity intervention

Three RCTs compared the timing of physical activity delivery, including comparison of physical activity during treatment and after treatment (Supplementary Table 8). There were no clear differences in pain outcomes by intervention timing in these studies [15, 49, 50].

### Physical activity setting or delivery method and pain outcomes

Eight RCTs compared physical activity delivery settings or approaches (Supplementary Table 8). For supervised compared to home-based or unsupervised activity, two studies found greater effects for supervised interventions [16, 21] while four studies found no difference [137, 140, 150, 156]. There were no clear differences for telehealth supported compared to self-directed interventions or for an oncologist exercise recommendation with or without a motivational package [109, 145].

#### GRADE

GRADE appraisal results are presented in Table 1. The quality of the evidence for an effect of physical activity on pain intensity, bodily pain, or pain interference in adults with cancer was initially graded as high, as results were based on meta-analysis of RCTs. However, these were then graded down to low, owing to high heterogeneity in effects between studies, possible publication bias, and wide confidence limits. The evidence for an effect of physical activity on pain sensitivity or analgesic use was graded as very low, owing to the low number of studies that prevented meta-analysis, as well as variation within these studies.

**Table 1:** GRADE evidence appraisal for physical activity and cancer pain outcomes

Outcome	Meta-analysis study N (participant N)	Meta-analysis effect estimate (SMD (95% CI))	Quality of evidence
Pain intensity	47 (3734)	− 0.30 (− 0.45, − 0.15)	Low^aa^
Bodily pain	19 (1170)	0.28 (0.01, 0.56)	Low^aa^
Pain interference	3 (207)	− 0.13 (− 0.42, 0.15)	Low^b^
Pain sensitivity	NA	NA	Very low^c^
Analgesic use	NA	NA	Very low^c^

^a^Graded down due to heterogeneity and publication bias

^b^Graded down due to low certainty and study sample size

^c^Graded very low as meta-analysis not possible

## Discussion

This review examined the effect of physical activity on pain outcomes in people who have or have had cancer. The review found that physical activity can reduce pain intensity and pain sensitivity in people with cancer. However, high heterogeneity does limit certainty of these findings.

The primary strength of this review is the inclusion of RCTs, RXTs, and prospective observational studies, thus synthesising evidence from multiple study designs. The review also synthesises evidence for numerous cancer types, physical activity types, and pain outcomes. It addresses clinically relevant questions that are central to the lived experience of most people with cancer, as well as many cancer survivors. This review also has several limitations that should be considered when interpreting the findings. First, high heterogeneity limits the certainty of the review findings. To an extent, high heterogeneity should be expected, given the range of cancer types and stages, as well as the range in timing, dose, and delivery setting of physical activity. Nonetheless, our ability to generalise this finding to all persons with cancer-related pain is limited. The review did not include children or adolescents or survivors of childhood cancers, and therefore, the findings only apply to adults. Few of the included studies assessed pain as a primary outcome, with most assessing it as a part of a broader quality of life assessment. This meant that while pain intensity was well measured, additional outcome domains recommended for pain trials, such as pain quality and affect, or analgesic use, were not widely captured. It also meant that few studies considered baseline pain levels in their inclusion criteria, and therefore, we cannot guarantee all participants included in the review were those that most need pain interventions. Further, as pain was not a primary outcome for many of the included studies, it is likely that the interventions delivered were not optimally designed for pain reduction. This may limit the potential benefit of observed effects relative to pain. Although the inclusion of prospective observational studies is a strength, as it facilitates triangulation of evidence and may provide a real-world perspective not offered by tightly controlled efficacy studies, several of these studies contained serious risk of bias and therefore provided only limited contribution to the overall findings.

That physical activity can reduce pain intensity as well as pain sensitivity in people who have or have had cancer is consistent with findings for other populations that experience pain. For example, healthy populations who are more active typically report less pain and have reduced sensitivity to painful stimuli than those who are less active [64, 141]. Further, physical activity has been shown to reduce pain and pain-related disability for people with conditions like back pain or arthritis [107, 134]. Physical activity has been proposed to decrease pain via central, biological, and psychosocial pathways [85, 134]. For people with cancer, additional factors that may underlie benefit include an improved ability to tolerate treatment or recovery from surgery, reduced cancer fatigue that could increase susceptibility to pain, or an increased perception of control, which can also improve perception of pain [85, 117].

Exercise type and delivery setting appeared to influence effect estimates. Subgroup meta-analyses showed stronger effects for resistance and/ or aerobic exercise than general physical activity and for supervised than unsupervised exercise. This may indicate increased benefit of exercise, which refers to more planned, structured, and intentional movement like lifting weights or doing continuous cardiorespiratory exercise at a set intensity, compared to broader physical activity, which refers to any type of movement performed by the muscles like working in a garden or walking for transport. It may also reflect that supervised exercise interventions are often delivered by exercise professionals, like physiotherapists or exercise physiologists, who may be more deliberate with their exercise prescription than people completing self-directed, unsupervised activity. This was not a finding in all individual studies and does not imply that self-directed physical activity is not beneficial or should not be recommended, as these interventions also saw a decrease in pain, and they may represent a more accessible or sustainable behaviour. However, it does suggest that an ideal physical activity intervention might include a supervised exercise component [25].

The evidence was stronger for breast cancer than other cancer types. This may be due to the larger number of studies that included women with breast cancer. It may also reflect the benefits of physical activity to the types of pain often experienced by women with breast cancer, such as post-surgery pain, lymphoedema pain, or arthralgia. Importantly, subgroup meta-analysis suggested likely pain improvement for multiple cancer types, and few individual studies documented increases in pain after physical activity. While further studies may be required to untangle the benefits of physical activity on pain-related to different types of cancer or treatments, our results support potential benefit for many people that experience cancer.

Future research should assess the recommended outcome domains for pain trials, which include pain intensity, pain quality, and pain impact on physical and mental function, as well as participant satisfaction [111]. Pending research questions to be investigated in future research could include how physical activity affects different types of pain experienced by people with cancer, what specific mechanisms underlie benefit, reasons for variation between patients, and how physical activity programmes should be delivered to people impacted by cancer-related pain. Clinically, these results support that physical activity can decrease pain in people with cancer and should be considered as a non-pharmacological option in cancer-related pain management guidelines. The findings do not have sufficient detail to recommend physical activity for many specific types of cancer-related pain; however, the findings do support the inclusion of physical activity in broader guidelines or patient facing materials [28].

## Conclusion

This review provides an overview for the evidence of an effect of physical activity on cancer pain. Physical activity interventions may decrease pain intensity and pain sensitivity in people living with and beyond cancer; however, high heterogeneity suggests that not all persons will respond the same way to all interventions. More research is needed to understand the specific effects of physical activity on different types of cancer-related pain.

### Supplementary Information

Below is the link to the electronic supplementary material.

Supplementary file1 (DOCX 905 KB)

## Data Availability

Data are available upon request.
