# Supplementary material for: Physical activity and pain in people with cancer: a systematic review and meta-analysis
Source: Support Care Cancer. 2024 Feb 6;32(3):145. doi: 10.1007/s00520-024-08343-3 (PMC10847204; doi:10.1007/s00520-024-08343-3)
Supplement: Supplementary file 1 — Supplementary file1 (DOCX 905 KB) [file 520_2024_8343_MOESM1_ESM.docx]

**Supplementary table 1: Search strategy for OVID Medline.** Performed on the 26^th^ of August 2021.

| **#** | **Search focus** | **Search terminology** | **Hits** |
| --- | --- | --- | --- |
| 1 | Publication Type (RCT’s and Cohort studies) | Clinical Trials as Topic (MH) OR Randomized Controlled Trial (MH) OR Cohort Studies (MH) OR Longitudinal Studies (MH) OR Prospective Studies (MH) OR Follow-Up Studies (MH) OR trial OR cohORt OR longitudinal OR prospective OR randomised OR randomized NOT (Comment OR editorial OR meta-analysis OR practice-guideline OR review OR letter (PT)) | 3,021,951 |
| 2 | Population (Cancer) | Neoplams (MH) OR Cancer OR Tumour OR Tumor OR carcinoma OR leukaemia OR lymphoma OR oncology | 3,271,004 |
| 3 | Intervention (MH) Exposure (Physical activity) | Preoperative Exercise (MH) OR Exercise (MH) OR Exercise Therapy (MH) Physical Therapy Modalities (MH) Physical Conditioning, Human (MH) OR Walking OR exercise OR “physical activity” OR “strength training” OR “resistance training” OR “aerobic training” OR “endurance training” | 476,444 |
| 4 | Outcome (Pain) | Pain Cancer Pain (MH) OR Pain Measurement (MH) OR Acute Pain (MH) OR Pain Threshold (MH) OR Breakthrough Pain (MH) OR Pain (MH) OR Pain Management (MH) OR Chronic Pain (MH) Arthralgia (MH) "Quality of Life" (MH) Analgesia OR Pain OR neuropathy OR neuralgia OR analgesia OR hypoalgesia | 1,025,490 |
| 5 | Combined | Search 1 AND 2 AND 3 AND 4 | 1,715 |
| 6 | Exclusion (animal studies) | Animals (MH) NOT Humans (MH) | 4,878,040 |
| 7 | Limit 1 | 5 NOT 6 | 1,709 |
| 8 | Limit 2 | Limit 7 to English | 1,673 |
| **Final** |  |  | **1,673** |

Key: MH = medical subject heading, PT = publication type. Title and abstract fields were searched for all keyword searches.

**Supplementary table 2: Study characteristics of randomised controlled trials**

| **Study name, first author, year** | **Participants** | **Supervision and setting** | **Intervention** | **Comparison** | **Outcome and outcome assessment** |
| --- | --- | --- | --- | --- | --- |
| Adams, 2018. | Male testicular cancer (stage I-IV) survivors, N = 52 (exercise = 29, control = 23), age ~44. | Supervised exercise performed in a rehabilitation facility. | Aerobic (HIIT) performed for 35 minutes, 3/ week, for 12 weeks. Included 4-minute work intervals at 75-95% VO2max. | Usual Care | Bodily pain assessed using the SF-36. |
| Ali, 2021. | Female breast cancer survivors with stage I-II lymphedema, N = 50 (aqua therapy = 25, land therapy = 25), age ~ 51. | Supervised exercise in a rehabilitation / hydrotherapy setting. | Aqua therapy or resistance training. Resistance training performed for 60 minutes, 3/ week, for 8 weeks. Aqua therapy group was the same as resistance training but performed in water. | Comparison of aqua and resistance exercise. | Pain intensity measured using a VAS scale. |
| Arbane, 2014. | People with NSCLC scheduled to receive surgery, N = 131 (exercise =64, control = 67), age ~68. | Combined supervised hospital and unsupervised home-based exercise | Combined aerobic and strength training for inpatients. Performed daily for 30 minutes following surgery. Home based exercise included 30 minutes of daily walking. | Usual care | Bodily pain assessed using the SF-36. |
| ASX Trial, Van Blarigan, 2023. | Participants with prostate cancer stage T2 or lower with no prior treatment for prostate cancer, N = 51 (exercise = 26, control = 25), age ~ 63 years | Home-based, unsupervised but supported via telehealth monitoring. | Aerobic exercise that included walking for 20-60-minutes, 3/ week, for 16-weeks. Intensity was from 45-85% of VO2max. | Control with printed exercise material. | Bodily pain assessed using the SF-36. |
| Backman, 2014. | People with stage I-IV breast or colorectal cancer with ongoing adjuvant therapy, N = 67 (exercise =34, control = 33), age ~54 | Supervised group walk and unsupervised exercise | Physical activity. Walking an encouraged 10,000 steps per day as well as a weekly group walking class. 10-week intervention. | Usual care | Pain intensity measured using the EORTC symptom scale |
| Bade, 2021. | People with stage III-IV NSCLC at any stage of treatment, N = 39 (exercise = 20, control =19), age ~65 | Unsupervised, real-world setting. | Physical activity promotion. Encouraged a daily step count increase of 400 steps per day above average. | Usual care | Pain intensity measured using the EORTC symptom scale |
| Barbosa, 2021. | Women with breast cancer and treatment related arthralgia, N = 60 (pilates = 20, circuit = 20, control =20), age ~54 | Unclear | Pilates or combined circuit training performed for 75 minutes, 2/ week, for 8 weeks. | Comparison of Pilates, circuit, and usual care. | Arthralgia pain intensity measured using a VAS and BPI. Pain medications. |
| Basen-Engquist, 2006. | Women with breast cancer who had completed their primary treatment within the last 7 years, N = 60 (exercise = 35, control = 25), age ~55 | Real-world physical activity. | Physical activity promotion. Promotion/ education meetings were conducted in a community/ church setting. | Usual care | Bodily pain measured using the SF-36. |
| BEAT Cancer program, Rogers 2009 (pilot study), 2016, & 2023 | Women who were survivors of stage I-IIIA breast cancer and receiving AI or estrogen receptor modulators, N = 222 (exercise = 110, control = 112), age ~ 54 | Combination of supervised and self-directed real world physical activity | Physical activity. Aim to increase physical activity to 150 minutes per week of moderate intensity walking. Conducted over 12-weeks. | Usual care | Pain intensity assessed using a 5-point Likert scale from the WOMAC.  Bodily pain measured using the SF-36. |
| Bland, 2019. | Women with stage I-III breast cancer scheduled to receive paclitaxel or docetaxel in 2 or 3 weekly cycles, N = 27 (during chemotherapy = 12, after chemotherapy = 15), age ~55. | Combination of supervised facility based and unsupervised home-based exercise. | Combined aerobic and resistance exercise performed for 25-40 minutes, 3/ week, for chemotherapy duration. | Comparison of exercise during compared to exercise after chemotherapy. | CIPN symptoms (including shooting or burning pain) measured with the on the EROTC CIPN20 subscale.  Temporal summation of pain using the pinprick test. |
| Bloomquist, 2019. | Women with stage I-III breast cancer, N = 153 (High = 75, Low = 78), age ~52. | Supervised group based and home-based exercise | High: combined aerobic and resistance exercise performed at low and high intensities for 12 weeks.  Low: Home based physical activity/ walking aimed at 10,000 steps per day | Combined compared to home-based activity | Pain related to lymphedema. NRS. |
| Boing, 2023. | Women with stage I-III breast cancer, N = 74 (Mat Pilates = 25, belly dance = 25, control = 24), age ~55. | Supervised. | Pilates or dance performed for 16-weeks. Pilates included mat Pilates with some resistance elements performed three times per week. Belly dance was performed to music in group settings three times per week. | Comparison of Pilates, belly dance, and control. | Pain assessed using a VAS. |
| Brocki, 2014. | People undergoing lung cancer surgery, N = 78 (intervention = 41, control = 37), age ~64-65. | Supervised and at home exercise | Combined aerobic and resistance training. One one-hour session a week that included warm up, aerobic, and resistance exercise. Both intervention and control completed at home exercise. | Supervised and at home exercise compared to at home exercise only. | Bodily pain measured using the SF-36. |
| Brown, 2018. | People with stage I-III colon cancer, N = 39 (low-dose = 14, high-dose =12, control = 13), age = 64% < 69yrs, 36%> 60yrs. | Home based exercise | Aerobic exercise. Low dose exercise included 150 minutes a week at 50-70% HRmax.  High dose exercise included 300 minutes per week at the same intensity. | Comparison of low dose, high dose, and usual care. | Bodily pain measured using the SF-36 |
| Brown, 2021. | Women with stage I-III breast cancer who had completed surgery, chemotherapy, or targeted therapy 6-months prior to study entry, N = 177 (exercise = 87, control = 90), age ~59. | Combined supervised and unsupervised exercise. | Combined aerobic and resistance exercise. Resistance performed 2/ week with dumbbells. Moderate intensity aerobic exercise was performed on 4-6/ week, up to 180-minutes per week for 52-weeks. Prescription guided by ACSM guidelines | Control advised to ask physician about exercise. | Bodily pain assessed using the SF-36. |
| CANEX (Cancer and Exercise Study), Adeline, 2021. | Men and women with curable cancer undergoing their first systemic treatment, N = 20 (exercise = 10, control = 10), age (range) 66 – 74. | Predominantly supervised exercise performed in a research lab setting. | Combined aerobic and resistance exercise performed 3/ week for 12 weeks. Aerobic exercise performed for 40 minutes at 75% HRR. Resistance training targeting major muscle groups and increasing in load. | Supervised stretching program. | Pain intensity assessed using the EORTC symptom scale |
| Cantarero-Villanueva, 2012, Alvarez-Salvago, 2023. | Women with stage I-IIIA breast cancer who had received a mastectomy or quadrantectomy with posterior breast reconstruction and completed co-adjuvant treatment, N = 65 (exercise = 32, control =33), age ~48 | Supervised exercise. | Aquatic/ water based physical therapy exercise including aerobic and resistance exercise performed for 1 hour, 3/ week, for 8 weeks. | Usual care | Pain intensity measured using a VAS.  Pain sensitivity using pressure pain thresholds |
| Cavalheri, 2017. | People with stage I-IIIA NSCLC who were 6-10 weeks post lobectomy or about to undergo post-surgery chemotherapy, N = 17 (exercise = 9, control = 8), age ~ 67 | Mainly supervised exercise sessions with some at home exercise | Aerobic and resistance exercise performed for 60 minutes, three times per week, for 8 weeks. | Usual activity | Bodily pain measured using the SF-36. |
| Chan, 2022. | Survivors of colorectal cancer who were considered disease free at enrolment, N = 42 (exercise = 21, control = 21), age ~55 years. | Unsupervised physical activity. | Physical activity, which was promoted with a Fitbit, daily text messages, and print education material. Activities encouraged included brisk walking, jogging, and resistance training. The intervention period was 12-weeks. | Usual care. | Bodily pain assessed using the SF-36. |
| Cheville, 2013. | People with stage IV lung or colorectal cancer, N = 56 (exercise = 26, control = 30), age ~65 | Home based exercise | Aerobic and resistance exercise. Aerobic exercise included self-directed walking. Resistance exercise targeted the upper and lower body and was performed four times. | Usual activity | Pain intensity assessed using a NRS. |
| CO-CUIDATE, Cantarero-Villanueva, 2017. | Survivors of colorectal cancer, N = 40 (exercise = 21, control = 19), age ~58-62 | Unclear | Aerobic and stability exercise performed 3/ week for 8/ weeks. Aerobic exercise lasted 25 minutes and included brisk walking, jogging, or running. | Usual care | Pain severity and pain interference assessed using the BPI.  Pain sensitivity assessed via pain pressure thresholds and algometry. |
| Cormie, 2013B. | Men with prostate cancer and established bone metastases, N = 20 (exercise = 10, control = 10), age ~ 72 | Supervised group-based exercise in an exercise clinic | Resistance exercise performed for 60 minutes, twice a week, for 12 weeks. Exercises targeted major muscle groups of the body. | Usual care | Bone pain intensity assessed using the Functional Assessment of Cancer Therapy Bone Pain Questionnaire.  Bodily pain measured using the SF-36. |
| Cormie, 2013C. | Women with breast cancer and breast cancer related lymphedema, N = 62 (high load = 22, low load = 21, control = 19), age ~ 57 | Supervised group exercise in an exercise clinic | Resistance exercise performed for 60 minutes, 2/ week, for 3-months. Resistance training was either high load or low load. | High load vs low load vs usual care | Pain severity measured using the BPI.  Pain interference measured using the BPI. |
| Cormie, 2015. | Men with prostate cancer scheduled to undergo ADT, N = 63 (exercise = 32, control = 31), age ~68. | Supervised group-based exercise in an exercise clinic | Aerobic and resistance exercise performed for 60 minutes, 2/ week, for 3 months. Aerobic exercise performed approximately 70-85% of HRmax. | Usual care. | Bodily pain measured using the SF-36. |
| Courneya, 2013 & 2014. | Women with stage I-III breast cancer initiating adjuvant chemotherapy, N = 301 (Standard aerobic = 96, High volume aerobic = 101, aerobic and resistance = 104), age ~50. | Supervised exercise. | Aerobic or aerobic and resistance training. Standard aerobic training targeted 150 mins of MVPA performed over 3 days per week. High aerobic training targeted 300 mins of MVPA over 3 days per week. Combined included standard aerobic as well as 3 days per week of strength training. | Comparison of standard aerobic, high aerobic and combined training. | Bodily pain measured using the SF-36. |
| Dhawan, 2020. | People with cancer receiving carboplatin and paclitaxel with chemo induced peripheral neuropathy, N = 45 (exercise = 22, control = 23), age ~ 51. | Unsupervised, home exercise program | Resistance exercise performed for 30 minutes, 7/ week, for 10 weeks. | Usual care | Neuropathic pain assessed by the Leeds Assessment of Neuropathic Symptoms and Signs pain scale |
| Dieli-Conwright, 2018. | Breast cancer survivors (stage 0-III) who were within 6-months of completing adjuvant treatment, n = 100, age ~54. | Supervised exercise in a research facility. | Combined aerobic and resistance exercise performed for 50-80 minutes, 3/ week, for 16 weeks. | Usual Care | Bodily pain measured using the SF-36. |
| Dimeo, 1997. | People with solid tumours who had completed some chemotherapy, N = 70 (exercise = 33, control = 37), age ~ 40 | Hospital based exercise (biking performed in bed). | Aerobic exercise with a bed ergometer. 1-minute blocks for a total of 30 minutes/day | Usual care? (unclear) | Pain intensity assessed using the WHO scale. |
| Ding, 2020. | People with malignant tumours on long-term chemotherapy, N = 98 (exercise = 49, control = 49), age ~52 | Unclear | Tai chi exercise performed for 60 minutes, 5/ week, during chemotherapy intermission. | Hand grip exercises. | Bodily pain assessed using the SF-36. |
| Do, 2015. | Women with stage 0-III breast cancer who had undergone radiation therapy, N = 62(early exercise = 32, delayed exercise = 30), age ~48. | Supervised exercise in a rehabilitation facility. | Combined aerobic and resistance exercise performed for 120 minutes, 5/ week, for 4 weeks. Aerobic exercise performed for 40 minutes at 40-75% VO2max. Strength exercise performed with TheraBands and balls. | Comparison of early exercise to delayed exercise. Delayed exercise received usual care for first 4-weeks. | Pain intensity assessed using the EORTC symptoms scale. |
| Dodd, 2010. | Women with breast, colorectal, or ovarian cancer who were set to begin their first chemotherapy treatment, N = 106 (exercise = 37, delayed exercise = 32, control = 37), age ~51. | Home based exercise | Aerobic exercise, performed for 20-30m minutes at 60-80%VO2max, 3-5/ week, | Comparison of timing of exercise prescription and usual care. | Worst pain intensity measured with a single item NRS. |
| Dong, 2019, 2020. | People with stage I-III breast cancer who had completed postoperative radiotherapy or chemotherapy, n = 50 (exercise = 26, control = 24) | Supervised (‘face to face televideo) | Combined aerobic and strength training. Aerobic training was performed 4/ week. Strength training performed 3/ week. 12 weeks. | Usual care | Bodily pain assessed using the SF-36 |
| EXERT trial. Arthuso, 2023 & Morielli 2023. | Male and females with rectal cancer scheduled to receive neoadjuvant chemoradiation therapy, N = 36 (exercise =18, control = 18), age ~ 57(12) years. | Supervised then unsupervised exercise. | Aerobic (HIIT) performed 3/ week for the duration of treatment. Session duration from 28-40 minutes at 85% of VO2max. After treatment, participants asked to complete >150 minutes of unsupervised moderate to vigorous aerobic activity per week. | Usual Care | Pain assessed on a 7-point scale. |
| Eyigor, 2018. | Women with breast cancer, N = 42 (Yoga group = 22, control group = 20), age ~52 | Supervised group yoga | Hatha Yoga performed 2/ week for 10 weeks. | Usual care | Shoulder and arm pain intensity at rest measured with the VAS. |
| Fields, 2016. | Women with breast cancer and treatment related arthralgia, N = 40 (exercise = 20, control = 20), age ~ 63. | Supervised and unsupervised all outdoors in parks | Aerobic Exercise: Nordic walking: Starting with 1 hour per week, ending with 4/30 minute sessions, 12 weeks total. | Usual care | Arthralgia worst pain, pain severity, and pain interference assessed using the BPI.  Bodily pain assessed using the SF-36.  Self-efficacy for managing pain measured with the PSEQ. |
| Galvao, 2010. | Men with prostate cancer undergoing ADT, N = 57 (exercise = 29, control = 28), age ~70. | Supervised group-based exercise. | Aerobic and resistance training performed 2/ week for 12 weeks. Aerobic exercise performed for 15-20 minutes at 65-80% HRmax. Resistance exercise targeted major muscle groups. | Usual care | Bodily pain measured using the SF-36. |
| Galvao, 2014. | Men with prostate cancer previously treated with ADT, N = 100 (exercise = 50, control = 50), age ~72. | Supervised exercise completed in exercise clinics as well as home-based exercise | Aerobic and resistance training performed 2/week for 12 months. Aerobic included 20-30 minutes at 70-85% HRmax. Resistance targeted major muscle groups. | Control group received a pedometer and PA information. | Bodily pain measured using the SF-36. |
| Garcia-Soidan, 2020. | Women who have had breast cancer and received surgery and chemotherapy in the previous six months, N = 316 (strength = 79, aqua = 79, aerobic = 79, control = 79), age ~63. | Supervised group-based exercise. | Resistance, aerobic, or aqua exercise performed for 60 minutes twice weekly.  Resistance exercise progressively increased in intensity. Aqua and aerobic exercise featured choregraphed exercises. | Comparison between strength, aqua, aerobic, and control. Control completed usual activity. | Bodily pain measured using the SF-36. |
| Griffith, 2009. | People with stage I-III solid tumours scheduled to receive chemotherapy or radiotherapy, N = 126 (exercise = 68, control = 68), age ~ 60 | Home based walking | Aerobic exercise. Brisk walking performed for 20-30 minutes at 50-70% HRmax, five times per week. | Usual activity. | MOS-pain subscale. |
| Hacker, 2017. | People scheduled to undergo hematopoietic stem cell transplantation, n = 67 (exercise = 33, control = 34), age ~53. | Combined supervised and unsupervised. | Resistance training performed 3/ week for 6-weeks. Training was moderate intensity and progressive. | Usual care and one-to-one education. | Pain intensity measured with the EORTC symptom scale. |
| Haines, 2010. | Women with newly diagnosed breast cancer undoing adjuvant therapy, N = 73 (exercise = 37, control = 36), age ~55. | Home based exercise | Resistance and aerobic exercise. Resistance exercise included body weight and hand weight exercises. Aerobic included 20 minutes of walking. Performed for 12-months. | Sham/ flexibility intervention control group | Pain intensity assessed with the EORTC symptom scale |
| Hammer, 2021. | People with breast, lung, gastrointestinal, or gynaecological cancer scheduled to undergo chemotherapy, n = 33 (exercise = 15, control = 18), age ~ 47-50. | Real world walking exercise. | Aerobic exercise that included walking performed for 30 minutes, 3/ week, performed for 6 months. | Control encouraged to walk regularly. | Pain intensity measured using a single-item NRS |
| Hayes, 2013. | Women with invasive breast cancer, n = 194 (face to face = 67, phone = 67, control = 60), age ~52 | Both supervised and home-based exercise | Combined aerobic and resistance training performed for 180 minutes per week over 4 sessions. – | Intervention delivered face to face vs intervention delivered via telephone vs usual care | Neuropathic pain measures as part of the Disabilities of the arm, shoulder, and hand questionnaire |
| Henke, 2014. | People with non-small cell lung cancer or small cell lung cancer in stage IIIA/IIIB/IV receiving inpatient palliative chemotherapy. N= 29 (exercise = 18, control = 11) Age unclear. | Supervised inpatient setting. | Aerobic and resistance training. Aerobic training was performed five days per week for 6 minutes at approximately 60% HRR. TheraBand resistance exercise performed every other day. | Usual care. | Pain intensity measured with the EORTC symptom scale. |
| Ho, 2016 & 2018 | Women with breast cancer who had undergone mastectomy or lumpectomy, awaiting radiation or in first week of radiation, N = 127 (exercise = 64, control = 63), Age ~49 | Supervised exercise in a hospital, community cancer support centre, and university setting | Dance movement therapy performed for 90 minutes, 2/ week, for 3 weeks. | Usual care | Pain severity and pain interference using the BPI |
| Hwang, 2008. | Women with breast cancer on a waiting list for radiotherapy, N= 37 (exercise = 17, control = 20), age ~46 | Supervised exercise | Combined aerobic and resistance exercise performed for 50 minutes, 3 times per week, for 5 weeks. | Usual care. | Pain intensity assessed using a VAS |
| Ibrahim, 2018. | Young women with stage I-III breast cancer, N = 59 (exercise = 29, control = 30), age ~39 | Supervised and unsupervised exercise. | Combined aerobic and resistance training. Performed 2-3/ week for 12 weeks. Intensity increased throughout. | Usual care | Pain severity and interference assessed using BPI-SF |
| IMPACT Study and YES Study, Cadmus, 2009. | Women with breast cancer about to start adjuvant treatment (N = 50) as well as breast cancer survivors (N=75). N = 125 (exercise = 62, usual care = 63), age ~55. | Home based exercise as well as supervised exercise | Home or supervised exercise. Specific exercise type unclear, although use of HR monitor suggests aerobic. | Usual care. | Bodily pain measured using the SF-36. |
| Irwin, 2015. | Breast cancer survivors using aromatase inhibitors for >6-months and with at least moderate pain, N = 121 (exercise = 61, control = 60), age ~61 | Supervised (at a local health club) and unsupervised (home/community based) exercise. | Combined aerobic and resistance training. Resistance training was supervised 2/ week. Aerobic training was self-directed and aimed to achieve 150 minutes per week. 12-month intervention duration. | Usual Care | Arthralgia worst pain, pain severity, and pain interference measured using the BPI.  Pain intensity measured using the WOMAC.  Analgesic use. |
| Jensen, 2014. | People with advanced gastrointestinal cancer with a life expectancy >6 months, N = 21(resistance = 11, aerobic = 10), age ~55. | Supervised exercise | Resistance or aerobic training performed for 45 minutes 2/week, for 12 weeks. Resistance training targeted large muscle groups. Aerobic training performed at 70-80% HRmax. | Resistance compared to aerobic training. | Pain intensity measured with the EORTC symptom scale. |
| Kang, 2022. | Women with stage I-III breast cancer, who received an implant-based reconstruction and post-operatively started chemotherapy or radiotherapy, N = 60 (exercise = 30, control = 30), age ~47 years. | Supervised exercise. | Combined aerobic and resistance exercise, performed for 1-hour, 3/ week, for 4-weeks. Exercise was supervised by a nurse and was consistent with the ACSM exercise guidelines. | The control group completed a 4-week exercise program supervised by a physical therapist. | Pain intensity assessed using the EORTC symptoms scales. |
| Ligibel, 2016. | Women with metastatic breast cancer with a life expectancy >12 months, N = 76 (exercise = 33, control = 43), age ~50. | Combined supervised and home exercise | Aerobic exercise that was moderate in intensity and targeted 150 minutes per week for 16 weeks. | Usual care | Pain intensity measured with the EORTC symptom scale. |
| Lin, 2021. | People with head and neck cancer undergoing chemotherapy and with a life expectancy > 6 months, N = 40 (exercise = 20, control = 20), age ~53. | Conducted at a university gym. | Aerobic and resistance training performed for 90 minutes, 3/ week, for 8 weeks. Moderate intensity intervention that included treadmill and elastic band exercises. | Usual care | Pain intensity measured with the EORTC symptom scale. |
| Lin, 2023. | People diagnosed with breast cancer and who had received surgery, N = 200 (aerobic exercise = 50, resistance exercise = 50, intensive follow up = 50, control/ joint mobility = 50), age ~50 years. | Remote supervision via WeChat. | Aerobic, resistance, or joint mobility exercise performed for 6-months. Aerobic exercise included 30-minutes of brisk walking at 60—80% of HR max, 5/ week. Resistance training performed with elastic bands 2-3/ day. Joint mobility included rehabilitation exercises performed 3/ day. | Aerobic compared to resistance compared to joint mobility with and without intensive follow up. | Pain intensity assessed using a numeric rating scale. |
| Litterini, 2013. | People with advanced/ terminal cancer, N = 66 (aerobic = 32, resistance = 34), age ~62. | Supervised exercise | Aerobic or resistance exercise performed for 30-60 minutes, 2/ week, for 10 weeks. Intensity influenced by participant tolerance and safety. | Aerobic compared to resistance exercise | Pain intensity measured with a VAS |
| LYCA, Ammitzboll, 2020. | Women with breast cancer post axillary lymph node dissection, N = 158 (exercise = 82, control = 76), age ~52. | Supervised and unsupervised | Resistance training performed 3/ week for 12-months. Load and intensity gradually increased. | Usual care | Presence of pain measured using a validated Danish Questionnaire  Pain intensity measured using an NRS.  Neuropathic pain measured using a Rasch validated scale |
| Machado, 2023. | People scheduled for surgery to treat stage I-IIIA lung malignancy, N = 41, (exercise = 20, control = 21), age ~68 years. | Home based with remote supervision via telephone. | Combined aerobic and resistance training. Aerobic included 30-minutes of walking performed 3/ week. Resistance training targeted lower body functional strength performed 2/ week. | Usual care | Pain intensity assessed using QLQ-C30 symptom scales. |
| Mardani, 2021. | Prostate cancer survivors, N = 71 (exercise = 35, control = 36), age ~69. | Combined supervise group and self-monitored exercise | Aerobic and resistance exercise starting at 60 minutes/ week and progressing to 150 minutes/ week for 12 weeks. | Usual care | Pain intensity measured with the EORTC symptom scale. |
| Messaggi-Sartor, 2019. | People with stage I or II NSCLC, N = 37 (exercise = 16, control = 21), age ~55. | Likely supervised | Aerobic and respiratory muscle exercise performed for 60 minutes, 3/ week, for 8 weeks. Moderate intensity aerobic exercise. | Usual care | Pain intensity measured with the EORTC symptom scale. |
| Moon, 2022. | Men with prostate cancer under active surveillance, N = 27 (exercise = 15, control = 12), age ~73 - 75 years. | Unsupervised home-based exercise. | Combined aerobic and resistance exercise. Aerobic included 30-min walking at moderate intensity, 5/ week. Resistance included body weight and resistance band exercise 3/ week. Participants also received a Fitbit. | Wait list control. | Bodily pain assessed with the SF-36. |
| Moraes, 2021. | Breast cancer survivors >6 months following treatment completion, N = 25 (exercise = 12, control = 13) age ~55. | Supervised exercise | Resistance training performed once weekly for 8 weeks. | Usual care (although unclear) | Bodily pain measured using the SF-36. |
| Nilsen, 2015. | Men with prostate cancer undergoing ADT, N = 58 (exercise = 28, control = 30) age ~66. | Supervised | Resistance training performed 3/ week for 16 weeks. | Usual activity | Pain intensity measured with the EORTC symptom scale. |
| Norris, 2015. | Men with non-metastatic prostate cancer, N = 30 (2-day = 14, 3-day = 16) age ~63. | Supervised at an exercise centre. | Resistance training performed 2 or 3 days per week for 12 weeks. Intensity progressed steadily. | 2 days compared to 3-day exercise. | Bodily pain measured using the SF-36. |
| Nyrop, 2017. | Women with breast cancer and AI-induced arthralgia, n = 62 (exercise = 31, control = 31), age ~53. | Real world exercise | Aerobic exercise that included walking 150 minutes per week for 6 weeks. | Usual activity control | Arthralgia related pain intensity assessed with a VAS as well as the WOMAC subscales |
| Odynets, 2018. | Breast cancer survivors who were post mastectomy and adjuvant therapy, N = 115 (water based 45, water and pilates = 40, yoga= 30), age (range) = 50-60. | Likely supervised. | Water aerobics, swimming, Pilates, and/ or Yoga. Participants completed 140 sessions over 12-months. | Water Aqua aerobics, swimming, and recreational aerobics  Vs  Swimming and Pilates  Vs  Yoga and stretching | Pain intensity assessed using a VAS.  Pain quality (including sensory, affective, and cognitive quality) assessed using McGill Pain Questionnaire. |
| OptiTrain breast cancer trial. Bolam, 2019 & Mijwel 2018A, 2018B, 2019. | Women scheduled to receive chemotherapy for stage I-III breast cancer N = 182 (RT-HIIT = 65, AT-HIIT = 60, control = 57), age ~53. | Supervised exercise in a clinic setting | Resistance training and HIIT, aerobic training and HIIT performed for 60 minutes, 2/ week, for 16 weeks. | Comparison of AT-HIIT, RT-HIIT, and usual care. | Pain sensitivity measured with pain pressure thresholds.  Pain intensity measured with the EORTC symptom scale. |
| PACES, vanWaart, 2015. | People with breast or colon cancer scheduled to undergo adjuvant chemotherapy, N = 197 (Ontrack = 71, onco-move =-62, control = 77), Age ~51 | Supervised or unsupervised (home-based) exercise. | Physical activity or combined aerobic and resistance training. Onco-move included 30 minutes, 5/ week of home-based physical activity. On-Track included 20 minutes of moderate to high intensity exercise performed 2/ week. Both interventions started with chemotherapy and lasted 3-weeks after completion. | Comparison of supervised, home-based, and usual care control. | Pain intensity measured using the EORTC symptom scale. |
| PACT Study (breast cancer), Travier, 2015. | Women with breast cancer and no distant metastases, N = 164 (exercise = 87, control = 77), Age (range) = 25 - 75 | Supervised exercise | Combined aerobic and resistance exercise, performed for 60-minutes, 2/ week, for 18-weeks. | Usual care. | Pain intensity measured using the EORTC symptom scale. |
| PACT Study (Colon cancer). Van Vulpen 2016. | People with recently diagnosed colon cancer scheduled to undergo chemotherapy, N = 33 (exercise = 17, control = 16), age ~ 58. | Supervised exercise | PACT trial. Combined aerobic and resistance exercise, performed for 60-minutes, 2/ week, for 18-weeks. | Usual care. | Pain intensity measured using the EORTC symptom scale. |
| PAM Study, Koevoets, 2022. | Women who were 2-4 years post diagnosis of stage I-III breast cancer, N = 181 (exercise = 91, control = 90), age ~52 years. | Supervised exercise. | Combined aerobic and resistance exercise. 4 hours/ week for 6-months. Aerobic exercise started at 40-60% of HRR and progressed to interval training. Resistance exercise included circuit training. | Wait listed control. | Pain assessed using EORTC symptom scales. |
| Park, 2015. | People with stage I-III colorectal or breast cancer who have completed primary and adjuvant treatment, N = 162 (exercise group 1 = 53, exercise group 2 = 50, control = 59), age ~51. | Self-directed, real-world exercise. | Physical activity and strength exercise. Participants a recommendation for 150 minutes of physical activity and twice weekly strength training. Some participants also received a motivational package. | Comparison of oncologist recommendation, oncologist recommendation and motivational package, and wait-list control. | Pain intensity measured with the EORTC symptom scale. |
| Pasyar, 2019. | Women with breast cancer related lymphedema, N = 27 (yoga = 12, control = 15) age ~51. | Predominantly supervised with some home-based yoga. | Yoga performed 3/week for 8 weeks. | Usual care control. | Pain intensity measured with the EORTC symptom scale. |
| Paulo, 2019. | Women with stage I-III breast cancer using AI. N = 36 (exercise = 18, control =18) age ~63 – 66. | Supervised exercise in an exercise setting. | Aerobic and resistance training performed for approximately 70 minutes, 3/ week, for 9-months. Intensity of exercises gradually increased throughout. | Control group performed 2/week stretching. | Arthralgia pain related to AI.  Bodily pain measured using the SF-36.  Pain intensity measured with the EORTC symptom scale. |
| Pelzer, 2023. | Males and females with non-metastatic breast or metastatic or non-metastatic prostate cancer who were between 6 – 52 weeks post primary treatment, N = 95 (aerobic training = 47, resistance training = 48), age ~55 – 61 years. | Supervised in a gym-like setting. | Aerobic or resistance training. Aerobic training included 30-minutes of moderate to vigorous cycling 2/ week or combined HIIT and continuous exercise. Resistance training targeted major muscle groups and was performed 2/ week. Program duration was 12-weeks. | Aerobic was compared to resistance training. | Pain assessed with EORTC symptom scales. |
| Peppone, 2015. | Breast cancer survivors who were between 2- and 24-months post-surgery, chemotherapy, and/or radiation therapy and currently receiving AI or TAM, N = 167 (yoga = 72, control = 95) age ~54. | Supervised group yoga. | YOCAS (Yoga for cancer survivors). Yoga performed for 75 minutes, 2/ week, for 4 weeks. | Standard care/ wait list control. | Pain intensity measured with a single item pain measure.  Pain presence measured with the FACIT-F. |
| Phys-Can Project, Ax, 2022. | Males and females with breast, colorectal, or prostate cancer scheduled to receive neoadjuvant oncological treatment, N = 577 (high intensity = 288, low to moderate intensity = 289), age ~ 58 years. | Supervised and unsupervised exercise. | Combined aerobic and resistance training for 6-months starting with the commencement of treatment. Aerobic included home based HIIT 2/ week  for the high intensity group or 150 minutes of walking or cycling type activities for the low intensity group. Resistance training was supervised 2/ week. | Comparison of high intensity with low to moderate intensity exercise. | Pain assessed using the EORTC |
| PhysSURG-B, Heiman, 2022. | Women with stage I-III breast cancer scheduled to undergo surgery, N = 286 (exercise = 139, control = 148), age ~63 years. | Unsupervised physical activity. | Aerobic physical activity, performed daily, 2-weeks before and 4-weeks after breast cancer surgery. Activity was moderate intensity. | Usual care with limited physical activity information. | Pain assessed using with the RAND-36. |
| Pope, 2018. | Women with stage 0-III breast cancer who completed primary treatment between 3months and 10 years ago, N = 20 (exercise = 12, control = 8) age ~53. | Self-directed activity performed in the real-world. | Physical activity promotion via the provision of a Polar watch and face book group support. | Control group could access the face book group and received training recommendations. | Pain intensity measured via PROMIS |
| Porserud, 2014. | People with urinary bladder cancer who underwent a radical cystectomy with ileal conduit, N = 10 (exercise = 4, control = 6) age ~72. | Supervised group exercise training in a hospital setting. | Aerobic and resistance training performed for 45 minutes, 2/ week, for 12-weeks. | Usual activity/ wait list control | Bodily pain measured using the SF-36. |
| Rastogi, 2020. | People with stage I-III colorectal or breast cancer that had completed their primary and adjuvant therapy, N = 50 (exercise = 26, control = 24), age ~54. | Self-directed physical activity in real world settings. | Physical activity. Participants were asked to increase their weekly MVPA to 150 minutes per week and daily steps to 10,000. | Usual activity | Bodily pain measured using the SF-36. |
| Reis, 2018. | Women with breast cancer who were undergoing chemotherapy and radiation therapy, N = 28 (exercise = 14, control =14) age ~46. | Supervised exercise. | Aerobic and resistance training performed for 60 minutes, 3/ week, for 12 weeks. | Usual treatment | Pain intensity and interference measured using the BPI |
| RHYTHM, Pisu, 2017. | Women with cancer who were 3 months post primary treatment completion, N = 29 (dance = 13, control = 16), age ~58. | Supervised dance lessons in one to one and group settings. | Dance were lessons conducted over 45 minutes, 1/week, for 12 weeks. Participants expected to practice at home. | Usual activity/ wait list control. | Bodily pain measured using the SF-36. |
| Rief, 2014. | People with cancer and bone metastases of the thoracic or lumbar segments of the vertebral column, N = 48 (exercise = 25, control = 23) age ~61-64, | Supervised exercises. | Resistance training performed for 30 minutes, 5/ week (treatment days), for 2 weeks. Exact exercise content differed according to patient presentation. | Respiratory exercises. | Pain intensity measured with a VAS |
| Schmidt, 2015. | Women with breast cancer undergoing adjuvant chemotherapy, N = 67 (resistance = 21, aerobic = 20, control = 26) age ~53-56. | Supervised exercise sessions | Aerobic or resistance training performed for 60 minutes, 2/ week, for 12 weeks. Intensity levels aligned with ACSM guidelines. | Comparison of aerobic, resistance, and usual care. | Pain intensity measured with the EORTC symptom scale. |
| Schmitt, 2016. | Breast cancer survivors who had completed chemotherapy or radiotherapy treatment, N = 28 (HIIT = 14, low/ moderate = 14) age ~54. | Supervised exercise sessions | HIIT or low to moderate aerobic exercise performed eight times over three weeks. HIIT included 8 x 1 minute hill sprints. Low/ moderate intensity exercise included 75 minutes of walking and cycling. | Comparison of HIIT to low/ moderate intensity aerobic exercise. | Pain intensity measured with the EORTC symptom scale. |
| Schumacher, 2021. | Men with prostate cancer undergoing ADT with or without concurrent radiation therapy, N = 115 (exercise = 72, control = 43) age ~68. | Supervised group exercises in an exercise clinic. | Aerobic and resistance exercise performed 2-3/ week for 6-months. Resistance training targeted major muscles and aerobic training worked towards 85% HRmax. | Usual activity | Pain intensity measured with the EORTC symptom scale. |
| Shobeiri, 2016. | Survivors of stage I-II breast cancer who had completed surgery, chemotherapy, or radiotherapy, N = 60 (exercise = 30, control = 30) age ~43. | Supervised group exercise. | Aerobic exercise, performed at a moderate intensity for 40-60 minutes, 2/ week for 10 weeks. | Usual activity | Pain intensity measured with the EORTC symptom scale. |
| Stigt, 2013. | People with non-small cell lung cancer scheduled to receive surgery, N = 49 (exercise = 23, control = 26), age ~63. | Supervised | Aerobic exercise performed at 60-80% VO2 max, 2/ week, for 4-weeks. | Usual care. | Bodily pain assessed using the SF-36.  Pain measured using the McGill pain questionnaire.  Analgesic medication use. |
| Sturgeon, 2022. | Females with non-metastatic breast cancer, N = 15 (exercise = 8, control = 7), age ~ 50 years. | Home based, unsupervised. | Aerobic exercise, which was supported with aerobics DVDs, telehealth support from an exercise professional, and heart rate monitoring. DVD activities ranged from walking to higher intensity aerobics. | Usual activity | Bodily pain measured with the SF-36. |
| Su, 2017. | People with stage II-IV head and neck cancer diagnosed within the past 6-months, N = 37(outpatient = 19, home based = 18), age ~48 | Combined supervised and at home. | Aerobic, anaerobic exercise performed at home or in an outpatient setting. Approximately 60 hours over 12-weeks. | Comparison of home based to outpatient exercise. | Pain intensity assessed using a VAS. |
| SUPPORT study, Steindorf, 2019. | People with non-retractable stage I-IV pancreatic cancer, N = 47 (supervised = 9, home-based = 21, control = 17) age ~59-63. | Supervised exercise or home-based exercise. | Resistance training performed for 60-minutes, 2/ week, for 6-months. Exercises targeted major muscle groups and intensity was directed by ACSM guidelines. | Comparison of home-based, supervised, and usual activity control. | Pain intensity measured with the EORTC symptom scale. |
| The ANTRAC Trial, Rasmussen, 2023. | Breast cancer survivors with self-reported pain more than 1.5 years after treatment completion, N = 20 (exercise = 10, control = 10), age ~ 60 years. | Supervised exercise. | Resistance exercise performed 2/ week for 12-weeks, with a gradual progression in intensity accompanied by a decrease in repetitions. | Habitual physical activity. | Pain sensitivity assessed via pressure thresholds. Pain intensity and pain frequency assessed using NRS for different body sites. Movement evoked pain assessed using an NRS after exercise sets. |
| The EFICANCER Randomized Clinical Trial, Mendizabal-Gallastegui, 2023. | Patients with stage IV gastrointestinal, breast, or non-small cell lung cancers receiving first-line chemotherapy, N = 90 (exercise = 47, control = 43, age ~57 years. | Supervised by a nurse. | Combined aerobic and resistance training, 3/ week for 2-months. Aerobic included 20-60 minutes at 40-60% HRR. Resistance included upper and lower body strength exercises. | Usual care. | Pain intensity assessed using EORTC symptom scales and bodily pain assessed using the SF-36. |
| The Fit-4-Home trial. Steffens, 2022. | Adults scheduled to undergo surgery for liver, stomach, or pancreatic cancer, N = 96, (exercise = 47, control = 49), age ~ 47-49 years. | Steps performed during inpatient stay with progress monitored by a physiotherapist. | Physical activity was promoted via provision of a Fitbit Charge and a daily step count calendar. Step goals were individualised, with participants encouraged to complete 50% more steps per day between baseline and hospital discharge. | Usual care. | Bodily pain assessed using the SF-36. |
| The PERFECT trial. van Vulpen 2021. | Participants with Oesophageal cancer who were 4-52 weeks post surgery, N = 120 (exercise = 61, control = 59), AGE | Supervised exercise in an outpatient setting. | Combined aerobic and resistance training. 60-minute sessions 2/ week that were individualised to the patients’ level of fitness. Participants also encouraged to be physically active for at least 30-minutes/ day. | Usual care | Pain assessed using EORTC symptom scales. |
| UK Prosper, Bruce, 2021. | Women with newly diagnosed non-invasive breast cancer scheduled for surgery and defined as high risk of upper limb disability, N = 382 (exercise = 191, usual care = 191), age ~58. | Supervised exercise with some non-supervised/ phone delivery possible. | Physiotherapy intervention that includes resistance exercise. Up to six sessions per participant, per year. Exercise progression based on joint decision between clinician and patient. | Usual care. | Acute, chronic, and neuropathic pain assessed using a numeric rating scale. |
| Vallerand, 2018. | Survivors of haematological cancer, N = 51 (self-directed = 25, telephone = 26), age ~ 53 | Unsupervised | Physical activity promotion. Participants received guidelines that encouraged increasing aerobic activity by 60 minutes per week and up to 300 minutes per week total. The telephone group received additional motivation/ counselling. | Comparison of telephone exercise to self-directed exercise. | Bodily pain assessed using the SF-36. |
| Vardar Yagli, 2015. | Breast cancer survivors who completed treatment >3 years ago, N = 40 (aerobic exercise = 21, aerobic and yoga = 19), age ~48 | Supervised exercise | Aerobic exercise or aerobic and yoga exercise. Aerobic exercise performed at 60-70% HR max for 30 minutes, 3/ week, for 6-weeks. Yoga program included an additional 60-minute yoga class performed 3/ week. | Comparison of aerobic only and aerobic with Yoga training. | Pain intensity measured using the EORTC symptom scale. |
| Weyhe, 2022. | Patients scheduled for surgery for pancreatic cancer, N = 75 (standard physiotherapy = 37, intensive physiotherapy = 38), age ~ 66 years. | Supervised and unsupervised exercise during inpatient and outpatient stay. | Intensive physiotherapy included aerobic, resistance, and physical activity that included bed cycling, walking, resistance band and dumbbell exercises. Outpatient physical activity included walking for up to 12-months follow up, encouraging step counts with a pedometer. | Standard physiotherapy. | Pain assessed using EORTC symptom scales. |
| Yang, 2021. | Patients with gastrointestinal cancer undergoing chemotherapy and considered high risk for depression, N = 80 (exercise = 40, control = 40), aged from 18 – 75 years. | Supervision status unclear. | Qigong exercise performed for 15-18-minutes, five times per week during the afternoon, for 4-weeks. Exercise imitated the movement and respiration of a monkey. | Usual care/ conventional therapy. | Pain assessed using EORTC symptom scales. |
| Zengin Alpozgen, 2017. | Women with stage I-III breast cancer and limited shoulder ROM, N = 55 (Pilates = 18, strength = 18, home = 19), Age ~50 | Supervised or home-based exercise. | Pilates, strength, or home-based exercise. Each session performed for 45 minutes, 3/ week, for 8 weeks. | Comparison of Pilates, light strength, and home-based exercise. | Pain intensity measured using a VAS. |
| Zhou, 2017. | Ovarian cancer survivors (stage I-IV) who completed chemotherapy >1-month ago, N = 113 (exercise = 61, control = 52), age ~57 | Unsupervised home-based exercise | Aerobic exercise/ physical activity. Participants encouraged to complete 150 minutes per week of brisk walking or other aerobic activity. Supported by phone calls and heart rate monitors. 6-month duration. | Usual care | Bodily pain assessed using the SF-36 |

**Key:** ACSM: American College of Sports Medicine, AI: Aromatase inhibitor, BPI: Brief Pain Inventory, CIPN: Chemotherapy Induced Peripheral Neuropathy, EORTC: European Organistion for Research and Treatment of Cancer, FACIT-F: Functional Assessment of Chronic Illness Therapy – Fatigue, HIIT: High intensity interval training, HR: Heart rate, HRR: Heart rate reserve, MOS-pain: Medical Outcomes Study Pain Measures, MVPA: moderate to vigorous physical activity, NRS: Numeric rating scale, PSEQ: Pain Self-Efficacy Questionnaire, SF-36: Short Form (36) Health Survey, VAS: Visual analogue scale, WHO: World Health Organisation, WOMAC: Western Ontario and McMaster Universities Osteoarthritis Index.

**Supplementary table 3: Study characteristics of randomised cross-over studies**

| Study name, first author, year | Participants | Setting | Intervention details | Comparison condition | Outcome and outcome assessment method |
| --- | --- | --- | --- | --- | --- |
| Bloomquist 2018 | Women undergoing chemotherapy for stage I-III breast cancer, N = 21. | Supervised gym-based exercise | Resistance training sessions that lasted for 30 minutes. Low load session was between 60-65% 1RM, high load was 85-90% 1RM | Heavy load compared to low load resistance training. Separated by a 7-day washout. | Pain related to lymphedema. NRS. |
| Clifford 2021 | Cancer survivors who had completed adjuvant chemotherapy or radiotherapy 3-12 months prior, N = 19. | Likely supervised lab-based exercise | Aerobic exercise. 30 minutes, 3/ week, for 2 weeks. High intensity performed at 60-70% HRR. Low intensity at 30-40% HRR. | Comparison of high and low intensity exercise, separated by a 6-week washout. | Pressure pain thresholds using algometry.  Bodily pain assessed using the SF-36 |
| Cormie 2013 A | Women with breast cancer and breast cancer related lymphedema, N = 17, age ~61 | Supervised exercise in an exercise clinic | Resistance exercise: 2 sets of 5 different exercises  High load group: 8-8 repetition maximum  Low load group: 15-20 repetition maximum  All participants completed 1 x session of high load and 1 x session of low load with a 10-12day wash out period in between | High load followed by low load vs low load followed by high load | Pain severity using VAS and BPI |
| Cormie 2016 | Women with breast cancer related lymphedema, N = 21, age ~62 | Supervised exercise. | Resistance training including low load, moderate load, and high load exercise. Each session contained the same upper body resistance exercises. | Comparison of Low, moderate, and high loads, separated by a washout period > 7 days | Lymphedema related pain assessed using a VAS. |
| Thomas 2020 | People with non-haematological cancer who had completed at least one cycle of chemotherapy and were scheduled to complete at least two more, N = 10 age ~51. | Supervised exercise during chemotherapy infusion. | Aerobic exercise performed for 20 minutes at 30-40% of HRR during chemotherapy infusion. | Comparison of aerobic to usual care. Washout period unclear but exercise/ usual care performed on consecutive chemotherapy sessions. | Pain intensity measured using ESAS symptom diaries. |
| Vanderbyl 2017 | People with advanced (stage III-IV) NSCLC or GI cancer scheduled to receive anti-cancer treatment and with a life-expectancy > 4 months, N = 19. | Supervised individual or group exercise in a hospital setting | Combined aerobic and resistance or medical Qigong. Combined exercise performed at 60-70% HRmax or 2-4 METS, with parameters tailored to individual abilities. Qigong conducted for 45 minutes and encouraged at home. | Comparison of Qigong to combined training separated by a 2-week washout period. | Pain intensity measured with a Likert scale. |

**Key:** BPI: Brief Pain Inventory, ESAS: Edmonton Symptom Assessment System, HRR: Heart rate reserve, MET: Metabolic equivalent of task, NRS: Numeric Rating Scale, NSCLC: Non-small cell lung cancer, RM: Maximum repetitions, SF-36: Short Form (36) Health Survey, VAS: Visual analogue scale, WHO: World Health Organisation, WOMAC: Western Ontario and McMaster Universities Osteoarthritis Index.

**Supplementary table 4: Study characteristics of observation studies**

| Study name, first author, year | Participants | Physical activity assessment method and comparison/ modelling | Outcome and outcome assessment method | Covariates adjusted for |
| --- | --- | --- | --- | --- |
| Belanger, 2011. | Young adult cancer survivors N=588, Age ~ 38.2yrs | The leisure score index from the leisure time questionnaire. | Bodily pain measured with the SF-36 | Age, sex, marital status, education, ethnicity, BMI, months since diagnosis, surgery, chemo, radiation, current treatment status, current cancer status, smoking, drinking, number of comorbidities |
| Branstrom, 2015. | Women who received surgery for breast cancer at select Stockholm hospitals, N = 750 | Self-reported physical activity. Undefined questionnaire. | Pain intensity measured using the EORTC Symptom scale. | Age, BMI, cancer stage, lymph node involvement, type of surgery, and type of adjuvant treatment |
| Cho, 2012. | Women with a diagnosis of breast, colorectal, or ovarian cancer who were about to commence their first cycle of chemotherapy, N = 199, age ~51 | Self-reported physical activity. Participants classified as exercisers if they met guidelines. | Single item NRS for worst pain intensity | Univariate analysis only. |
| Dore, 2022. | Women who had completed treatment for stage I-III breast cancer, N = 199, age ~55 | Actigraph GT3X accelerometers | Pain scored between 0-6 based on the number of pain symptoms in anatomical sites, pain experienced during sexual intercourse, headaches, or chest pain. | Age, education level, income, marital status, breast cancer stage, time since treatment, height and weight or BMI. |
| Flowers, 2021. | Women with breast cancer scheduled to receive surgery, N = 259, age ~56 years. | Physical activity level, categorised as sedentary, light physical activity, moderate physical activity, or hard physical activity. Assessment method was unclear. | Persistent pain assessed using the Breast cancer Pain Questionnaire. Neuropathic pain assessed using the NeuPPS. Pain catastrophising assessed using the Pain Catastrophizing Scale. | Univariate analysis. |
| HEAL Study, Alfano, 2007, & Forsythe, 2013. | Female breast cancer survivors who were stage I-IIIA, N = 545, age ~58 | Modifiable Activity Questionnaire | Pain and physical sensations assessed using 14 items from a previous study of breast cancer patients.  Bodily pain measured using the SF-36 | Age, education, race, stage of disease, treatment, menopausal status, smoking status, BMI, comorbidities |
| Klein, 2021B. | Women who completed surgery for breast cancer, N = 188, age ~52 | Self-reported physical activity using unnamed questionnaire. | Pain assessed using a single item NRS | Univariate analysis only. |
| Phillips, 2020. | Women with stage I-III breast cancer scheduled to receive chemotherapy, N = 67, age ~49 | Actigraph GT3X accelerometers | Symptom rating prompts scored 0-10. | Time and treatment status, age, BMI, comorbidities, health status, disease stage, chemotherapy type, day, and treatment number. |
| Sande, 2014. | People scheduled to receive radiotherapy for cancer induced bone pain that was secondary to bone metastases, N = 42, age ~64. | ActivPAL accelerometers. | Worst pain, average pain, and pain interference measured with the BPI. | Univariate analysis only. |
| Solberg Nes, 2012. | Lung cancer survivors, N = 1,937, age ~65. | Self-reported physical activity and physical activity intentions. | Pain frequency and severity measured with the LASA. | Stage at diagnosis and smoking status. |
| TOPCOP2,  Feng, 2023. | Men with metastatic prostate cancer starting their first line of treatment, N = 47, age ~ 75 years. | Physical activity assessed via smartphone app (e.g., Google Health, Fitbit, or Apple Health) to track daily step counts. | Pain assessed using the Edmonton Symptom Assessment System. | Age, frailty, and treatment coherence. |
| Tuomi, 2023. | Patients diagnosed with head and neck cancer and receiving curative treatment, N = 49, age ~ 64 years. | Self-reported physical activity was assessed using the Saltin-Grimb PA level scale. Objective physical activity was assessed using Axivity AX3 accelerometers. | Pain was assessed using EORTC symptoms scales. | Baseline health related quality of life. |
| Zhuang, 2023. | Patients with newly diagnosed lung cancer, N = 367, age ~ 60 years. | Self-reported physical activity collected with the IPAQ-Long. | Pain and pain medication assessed using EORTC symptom scales. | Age, sex, education, BMI, smoking history, alcohol consumption, cancer stage, tumour diameter, and therapy method. |

**Key: BMI: Body mass index,** BPI: Brief Pain Inventory, EORTC: European Organistion for Research and Treatment of Cancer, IPAQ-Long: International physical activity questionnaire – long form, LASA: Single-item linear analogue scale assessments, NRS: Numeric rating scale, NeuPPS: Neuropathic Pain Scale for Postsurgical Patients, SF-36: Short Form (36) Health Survey.

**Supplementary Table 5: Risk of Bias in Randomised Control Trials**

| **Study** | **Risk of bias item** | | | | | | |
| --- | --- | --- | --- | --- | --- | --- | --- |
|  | **1a**  **Selection bias (random sequence generation)** | **1b**  **Selection bias (allotment concealment)** | **2**  **Performance bias** | **3**  **Detection bias** | **4**  **Attrition bias** | **5**  **Reporting bias** | **6**  **Other bias** |
| Adams, 2018 | Low | Low | High | Low | Low | Low | Low |
| Adeline, 2021 (Canex Study) | Low | Low | High | Low | High | Low | Low |
| Ali 2021 | Low | Low | High | Low | Low | Low | Low |
| Ammitzboll 2020 | Low | Low | High | Low | Low | Low | Low |
| Arbane 2014 | Low | Low | High | Low | High | Low | Low |
| Arthuso 2023 (EXERT trial) | Low | Low | High | Low | High | Low | Low |
| Ax 2022 (Phys-Can project) | Low | Low | High | Low | High | Low | Low |
| Backman 2014 | Low | Low | High | Low | High  Low attrition but low percentage meeting step goals. | Low | Low |
| Bade 2021 | Low | Low | High | Low | Low | Low | Low |
| Barbosa 2021 | Low | Low | High | Low | High | Low | Low |
| Basen-Engquist 2006 | Low | Low | High | Low | Low | Low | Low |
| Bland 2019 | Low | Low | High | Low | High | Low | Low |
| Bloomquist 2019 | Low | Low | High | Low | High | Low | Low |
| Boing 2023 | Low | Low | High | Low | High | Low | Low |
| Bolam 2019, Mijwel 2018-2019 (Opti-Train) | Low | Low | High | Low | High | Low | Low |
| Brocki 2014 | Low | Low | High | Low | High | Low | Low |
| Brown 2018 | Low | Low | High | Low | Low | Low | Low |
| Brown 2021 | Unclear | Low | High | Low | High | Low | Low |
| Bruce 2021 (UK Prosper) | Low | Low | High | Low | High | Low | Low |
| Cadmus 2009  (IMPACT Study and YES Study) | Low | Low | High | Low | High  Low attrition but low percentage meeting activity time goal. | Low | Low |
| Cantarero-Villanueva 2012 | Low | Low | High | Low | Low | Low | Low |
| Cantarero-Villanueva 2017 | Low | Low | High | Low | High | Low | Low |
| Cavalheri 2017 | Low | Low | High | Low | Low | Low | Low |
| Chan 2022 | Unclear | Unclear | High | Low | Low | Low | Low |
| Cheville 2013 | Low | Low | High | Low | High | Low | Low |
| Cormie 2013 B | Low | Low | High | Low | Low | Low | Low |
| Cormie 2013 C | Low | Low | High | Low | Low | Low | Low |
| Cormie 2015 | Low | Low | High | Low | Low | Low | Low |
| Courneya 2013, 2014 | Low | Low | High | Low | High  Low attrition but low percentage meeting minutes prescription | Low | Low |
| Dhawan 2020 | Low | Low | High | Low | Low | Low | Low |
| Dieli-Conwright 2018 | Low | Low | High | Low | Low | Low | Low |
| Dimeo 1997 | Unclear | Unclear | High | Low | Low | Low | Low |
| Ding 2020 | Unclear | Unclear | High | Low | Low | Low | Low |
| Do 2015 | Low | Unclear | High | Low | High | Low | Low |
| Dodd 2010 | Unclear | Unclear | High | Low | High | Low | Low |
| Dong 2019, 2020 | Unclear | Unclear | High | Low | High | Low | Low |
| Eyigor 2018 | Unclear | Unclear | High | Low | High | Low | Low |
| Fields 2016 | Low | Low | High | Low | Low | Low | Low |
| Galvao 2010 | Low | Low | High | Low | Low | Low | Low |
| Galvao 2014 | Low | Low | High | Low | High | Low | Low |
| Garcia-Soidan 2020 | Low | Low | High | Low | Low | Low | Low |
| Griffith 2009 | Unclear | Unclear | High | Low | Low | Low | Low |
| Hacker 2017 | Low | Low | High | Low | High | Low | Low |
| Haines 2010 | Low | Low | High | Low | High | Low | Effect on outcomes -bias from patients starting other forms of exercise |
| Hammer 2021 | Low | Low | High | Low | High | Low | Low |
| Hayes 2013 | Low | Low | High | Low | High | Low | Low |
| Heiman 2022 (PhysSURG-B) | Unclear | Unclear | High | Low | High | Low | Low |
| Henke 2014 | Unclear | Unclear | High | Low | Low | Low | Low |
| Ho 2016, 2018 | Low | Low | High | Low | High | Low | Low |
| Hwang 2008 | Unclear | Unclear | High | Low | Low | Low | Low |
| Ibrahim 2018 | Low | Low | High | Low | Low | High  Results for each group not presented | Low |
| Irwin 2015 | Low | Low | High | Low | High | Low | Low |
| Jensen 2014 | Unclear | Unclear | High | Low | High | Low | Low |
| Kang 2022 | Low | Low | High | Low | Low | Low | Low |
| Koevoets 2022 (PAM Study) | Low | Low | High | Low | Low | Low | Low |
| Ligibel 2016 | Low | Low | High | Low | High | Low | Low |
| Lin 2021 | Low | Low | High | Low | High | Low | Low |
| Lin 2022 | Low | Low | High | Low | High | Low | Low |
| Litterini 2013 | Low | Unclear – personnel aware | High | Low | Unclear – not reported | Low | No control intervention |
| Machado 2023. | Low | Low | High | Low | Low | Low | Low |
| Mardani 2021 | Low | Low | High | Low | Low | Low | Low |
| Mendizabal-Gallastegui, 2023 (EFICANCER RCT) | Low | Low | High | Low | High | Low | Low |
| Messaggi-Sartor 2019 | Low | Low | High | Low | High | Low | Low |
| Moon 2022 | Unclear | Unclear | High | Low | High | Low | Low |
| Moraes 2021 | Unclear | Unclear | High | Low | Low | Low | Low |
| Nilsen 2015 | Low | Low | High | Low | High | Low | Low |
| Norris 2015 | Low | Low | High | Low | Low | Low | Low |
| Nyrop 2017 | Unclear | Unclear | High | Low | High | Low | Low |
| Odynets 2018 | Unclear | Unclear | High | Low | Unclear | Low | Low |
| Park 2015 | Low | Low | High | Low | High | Low | Low |
| Pasyar 2019 | Low | Low | High | Low | High | Low | Low |
| Paulo 2019 | Unclear | Unclear | High | Low | High | Low | Low |
| Pelzer 2023. | Unclear | Unclear | High | Low | High | Low | Low |
| Peppone 2015 | Low | Unclear | High | Low | Unclear | Low | Low |
| Pisu 2017  (RHYTHM) | Unclear | Unclear | High | Low | Low | Low | Low |
| Pope 2018 | Low | Low | High | Low | High | Low | Low |
| Porserud 2014 | Low | Low | High | Low | High | Low | Low |
| Rasmussen, 2023 (The ANTRAC Trial) | Low | Low | High | Low | Low | Low | Low |
| Rastogi 2020 | Low | Low | High | Low | Low | Low | Low |
| Reis 2018 | Low | Unclear | High | Low | Low | Low | Low |
| Rief 2014 | Low | Low | High | Low | Low | Low | Low |
| Rogers 2016  (BEAT Cancer) | Low | Low | High | Low | Low | Low | Low |
| Schmidt 2015 | Low | Low | High | Low | High | Low | Low |
| Schmitt 2016 | Low | Unclear | High | Low | Low | Low | Low |
| Schumacher 2021 | Low | Low | High | Low | Low | Low | Low |
| Shobeiri 2016 | Low | Low | High | Low | High | Low | Low |
| Steffens 2022 (The Fit-4-Home trial) | Low | Low | High | Low | Low | Low | Low |
| Steindorf 2019  (SUPPORT Study) | Low | Low | High | Low | High | Low | Low |
| Stigt 2013 | Low | Unclear | High | Low | High | Low | Low |
| Sturgeon 2022 | Unclear | Unclear | High | Low | Low | Low | Low |
| Su 2017 | Low | Unclear | High | Low | Low | Low | Low |
| Travier 2015  (PACT Study) | Low | Low | High | Low | High | Low | Low |
| Vallerand 2018 | Low | Low | High | Low | Low | Low | Low |
| van Vulpen 2021 (The PERFECT trial) | Low | Low | High | Low | Low | Low | Low |
| Van Blarigan 2023 (ASX Trial) | Low | Low | High | Low | Low | Low | Low |
| Van Vulpen 2016 (PACT Study) | Low | Low | High | Low | High | Low | Low |
| vanWaart 2015 (PACES) | Low | Unclear | High | Unclear | High | Low | Low |
| VardarYagli 2015 | Low | Low | High | Low | High | Low | Low |
| Weyhe 2022 | Low | Low | High | Low | High | Low | Low |
| Yang 2021 | Low | Low | High | Low | Low | Low | Low |
| ZenginAlpozgen 2017 | Low | Unclear | High | Low | Low | Low | Low |
| Zhou 2017 | Low | Low | High | Low | High | Low | Low |

**Supplementary Table 6: Risk of Bias in Randomised Cross-Over Trials (Cochrane risk of bias tool for randomized trials (RoB 2)**

| **Study** | **1**  **Bias arising from randomisation** | **2**  **Bias arising from period and carryover effects** | **3**  **Bias due to deviations from intended intervention** | **4**  **Bias due to missing outcome data** | **5**  **Bias in the measurement of the outcome** | **5**  **Bias in selection of the reported results** |
| --- | --- | --- | --- | --- | --- | --- |
| Bloomquist 2018 | Low | Low | Low | Low | Low | Low |
| Clifford 2021 | Low | Low | Low | Low | Low | Low |
| Cormie 2013 | Low | Low | Low | Low | Low | Low |
| Cormie 2016 | Low | Low | Low | Low | Low | Low |
| Thomas 2020 | Low | High | Low | Low | Low | Low |
| Vanderbyl 2017 | Low | Low | High | Low | Low | Low |

**Supplementary Table 7: Risk of Bias in Observation Studies**

| **Study Cohort** | **Confounding** | **Participant selection** | **Exposure classification** | **Exposure departure** | **Missing data** | **Outcome Assessment** | **Reporting Results** | **Overall risk of bias** |
| --- | --- | --- | --- | --- | --- | --- | --- | --- |
| Alfano 2007 (HEAL Study) | Moderate | Low | Moderate | Moderate | Low | Low | Low | Moderate |
| Belanger 2011 | Moderate | Low | Moderate | Moderate | Low | Low | Low | Moderate |
| Branstrom 2015 | Moderate | Low | Moderate | Moderate | Low | Low | Low | Moderate |
| Cho 2012 | Serious | Low | Moderate | Moderate | Low | Low | Low | Serious |
| Dore 2021 | Moderate | Low | Low | Moderate | Low | Low | Low | Moderate |
| Feng 2023 (TOPCOP2) | Serious | Low | Moderate | Moderate | Low | Low | Low | Serious |
| Flowers, 2021 | Serious | Low | Serious | Moderate | Low | Low | Low | Serious |
| Klein 2021 B | Serious | Low | Moderate | Moderate | Low | Low | Low | Serious |
| Phillips 2020 | Moderate | Low | Low | Moderate | Low | Low | Low | Moderate |
| Sande 2014 | Serious | Low | Low | Moderate | Low | Low | Low | Serious |
| Solberg Nes 2012 | Serious | Low | Moderate | Moderate | Low | Low | Low | Serious |
| Tuomi 2023 | Serious | Low | Low | Moderate | Low | Low | Low | Serious |
| Zhuang 2023 | Moderate | Low | Moderate | Moderate | Low | Low | Low | Moderate |

**Supplementary Figure 1: Physical activity and pain intensity by cancer site**

**
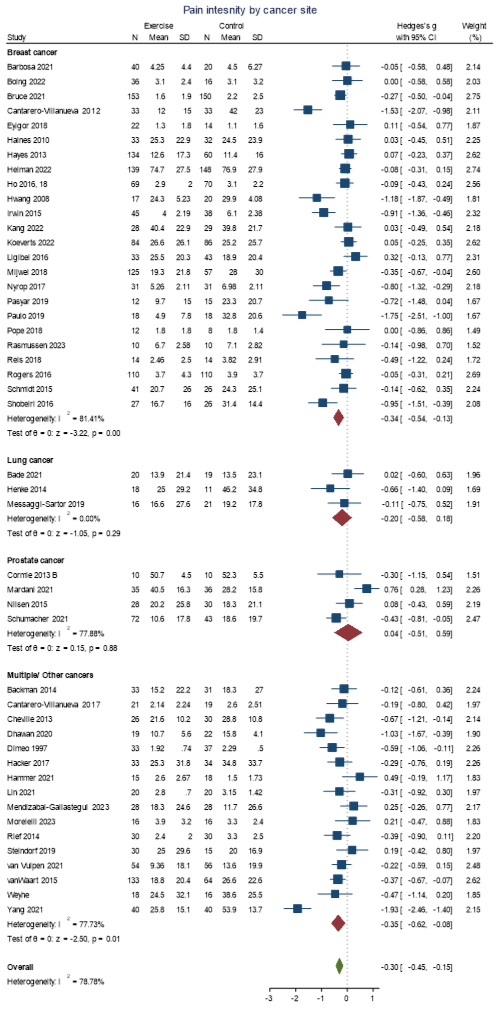
**

**Supplementary Figure 2: Physical activity and pain intensity by physical activity type**

**
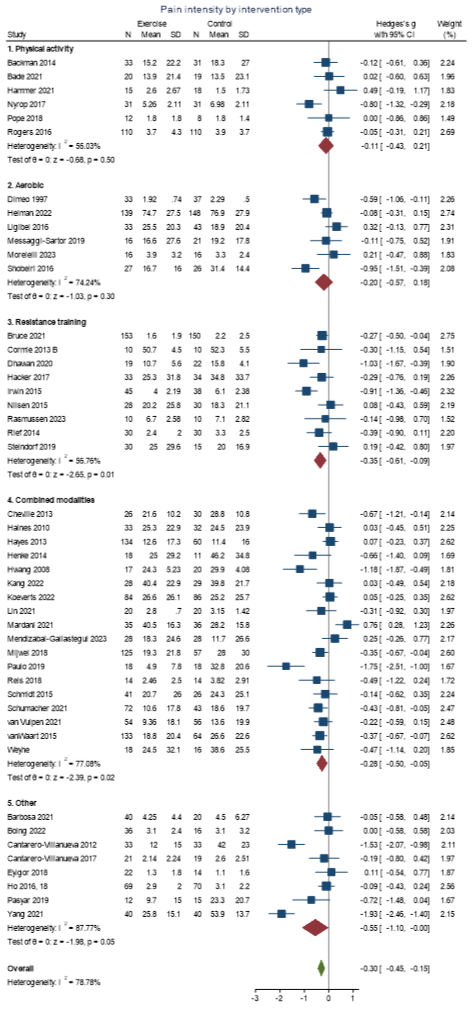
**

**Supplementary Figure 3: Physical activity and pain intensity by supervision status**

**
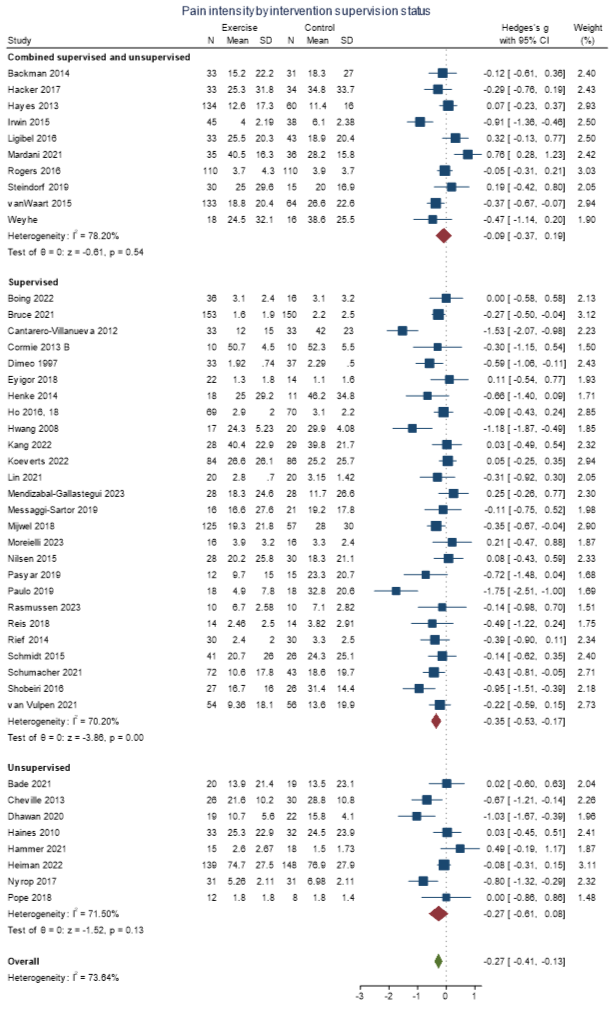
**

**Supplementary Figure 4: Physical activity and bodily pain by cancer site**

**
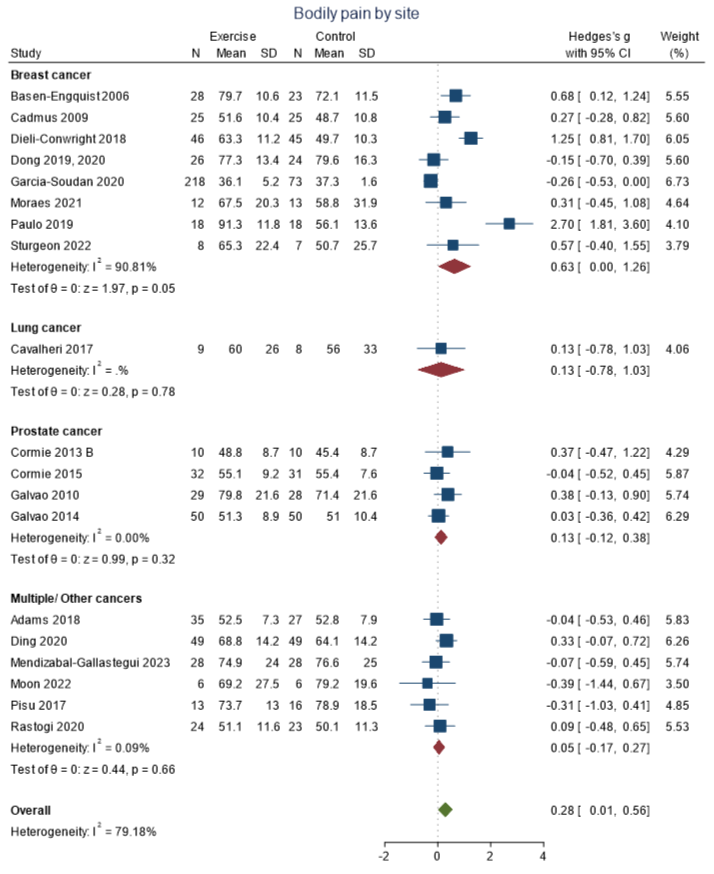
**

**Supplementary Figure 5: Physical activity and bodily pain by physical activity type**

**
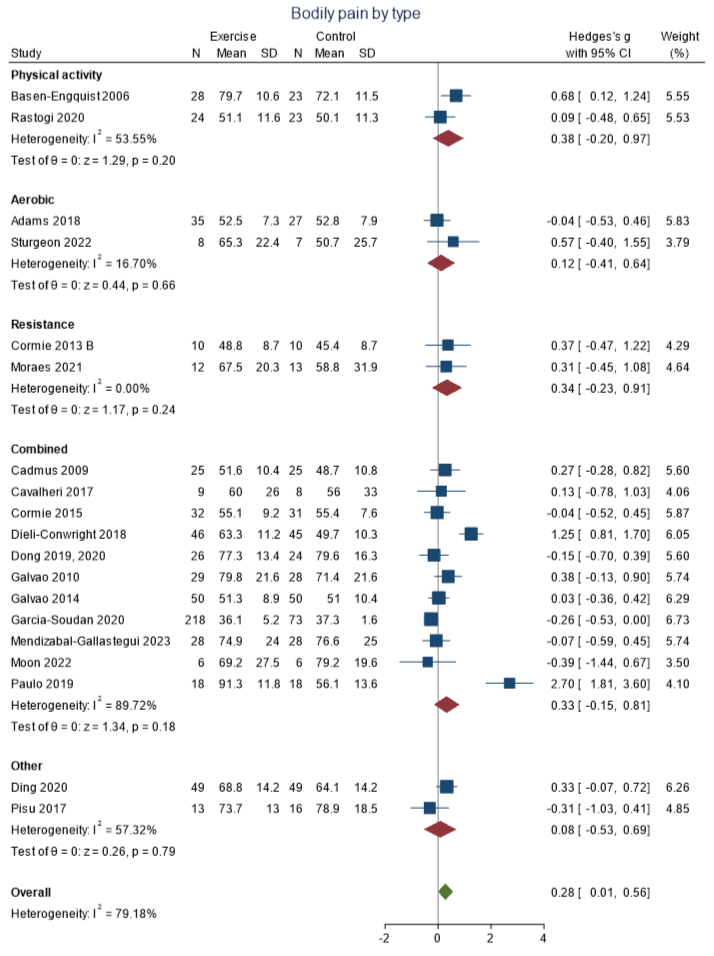
**

**Supplementary Figure 6: Physical activity and bodily pain by supervision status**

**
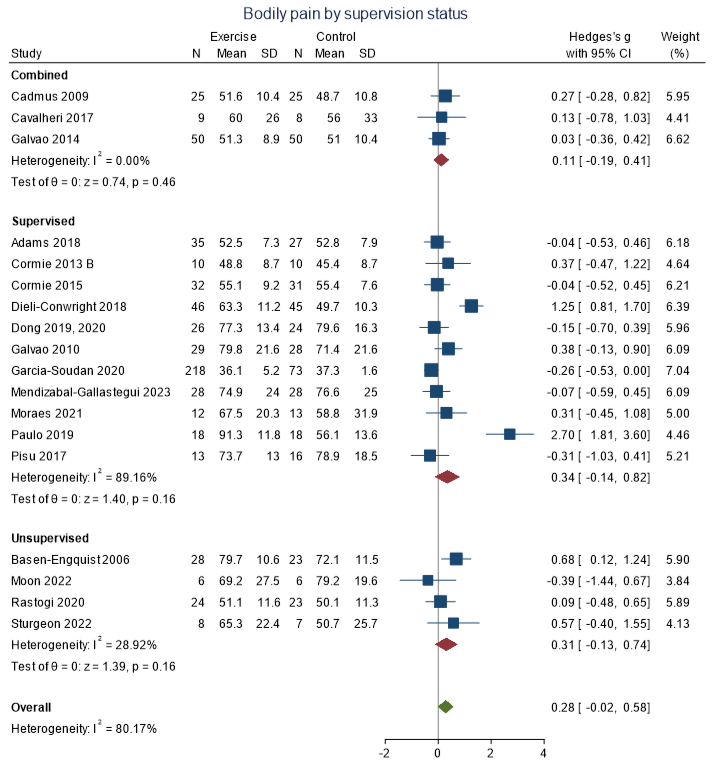
**

**Supplementary figure 7A-C. Funnel plots indicating potential publication bias.**


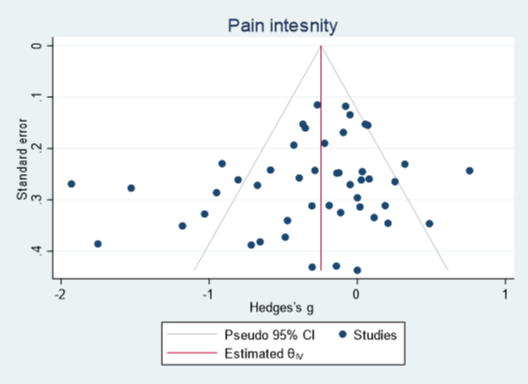

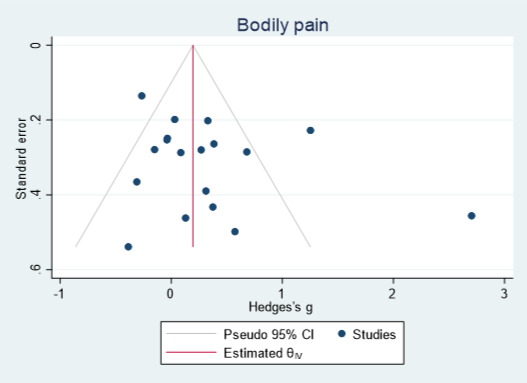


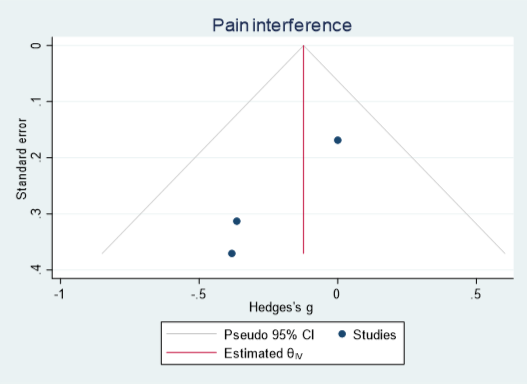


**Table 8. Results from individual intervention studies not included in the meta-analysis**

| **Pain intensity** | |
| --- | --- |
| Barbosa 2021 | Greater decreases in pain for the Pilates compared to circuit group. |
| Cormie 2013 | Pain intensity decreased following low or high load resistance training but not following control. Differences were not significant. |
| Lin 2023 | Pain levels decreased in all participants over time. Joint mobility, aerobic exercise, and resistance exercise or intensive follow up showed the greatest decrease in pain. |
| Machado 2023 | Pain intensity was lower following lung cancer surgery in people who received aerobic and resistance training (median = 8.5 IQR = 0 – 17) compared to usual care (median = 33 IQR = 8.5 – 50). |
| Park 2015 | Pain decreased more in participants who received an oncologist recommendation to exercise (change = -6.60) and those received recommendation and a motivational package (change = -7.00) compared to control (change = -2.26). |
| Travier 2015 | Small decrease in pain following the exercise intervention compared to control. Between group mean difference = -3.0 (-9.4, 3.5). Effect size = -0.12. Decrease not maintained 18-weeks post intervention. |
| Van Vulpen 2016 | No difference in pain between intervention and control groups. Between group mean difference = -0.6 (-17.1, 15.9). Effect size = -0.03. |
| **Bodily pain** | |
| Alfano 2007 | Moderate to vigorous physical activity, vigorous activity, household activity, and sports or recreational activity not related to bodily pain. |
| Arbane 2014 | No difference in pain changes between groups. Mean change difference = 3 (-13.3, 19.5). |
| Belanger 2011 | Less sedentary time and more physical activity associated with higher (i.e., better) bodily pain. |
| Brown 2018 | Pain increased more in the control group than low (MD = 3.24+/-7.12) or high (MD = 8.01+/-7.38) dose aerobic groups, but these differences were not significant. |
| Brown 2021 | No difference in pain changes between groups. Intervention effect = -0.31 (-5.54, 4.93). |
| Chan 2022 | No difference in pain changes between groups. Intervention effect = -2.3 (-14.3, 9.6) |
| Clifford 2021 | No change in bodily pain was reported after 2-weeks of high or low intensity exercise. |
| Cormie 2013 | No differences in bodily pain for control, low load, and high load resistance training (p = 0.4). |
| Porserud 2014 | Bodily pain improved following the intervention (Mean change = 27.7+/-20.9) but not control (Mean change = 2.9+/-39.2) |
| Steffans 2022 | No difference in pain changes between a step count intervention and usual care. |
| Van Blarigan 2023 | No change in pain following a home-based walking program relative to control. |
| Zhou 2017 | No difference between exercise or control. Effect mean = 1.9 (-1.5, 5.3). |
| **Pain sensitivity** | |
| Cantarero-Villanueva 2012 | Pain pressure thresholds increased (i.e., pain sensitivity decreased) following aquatic exercise for affected joints in the neck but not affected shoulder muscles or affected and unaffected metacarpals. Between group mean differences ranged from -2.8kPA (-45.3, 39.6) to 27.7kPA (3.9, 50.4). |
| Cantarero-Villanueva 2017 | Pain pressure thresholds increased (i.e., pain sensitivity decreased) following aerobic and stability exercise for sites near the lumbar spine and some but not all abdominal wall sites. There was no change in pain sensitivity at the 2^nd^ metacarpal. Between group mean differences ranged from 23.95kPA (-38.5, 86.4) to 182.25kPA (85.1, 279.4). |
| Clifford 2021 | Rectus femoris (exercising muscle): A single bout of exercise decreases pain sensitivity with a difference between intensities (MD = -0.51kg/cm^2^ SE = 0.15). High intensity exercise elicits a moderate size pain sensitivity reduction, low intensity exercise elicits a small reduction. A short training period (2-weeks) elicits a decrease in post exercise pain sensitivity, with no difference between training intensities (MD = 0.01 kg/cm^2^ SE = 0.25).  Biceps brachii (non-exercising muscle): A single bout of exercise did not change pain sensitivity. A short period of training decreased pain sensitivity in biceps brachii, with a non-significant difference between intensities (MD = -0.20kg/cm^2^ SE = 0.14). |
| Mijwel 2018 | Trapezius muscle: Resistance training and HIIT increased pain pressure thresholds (i.e., pain sensitivity decreased) compared to usual care (ES = 1.17) and aerobic training and HIIT (ES = 0.45). Aerobic training and HIIT also increased pain pressure thresholds compared to control (ES = 0.45).  Gluteal muscle: Resistance training and HIIT increased pain pressure thresholds (i.e., pain sensitivity decreased) compared to usual care (1.02). Aerobic training and HIIT also increased pain pressure thresholds compared to control (ES = 0.61). There was no difference between resistance training and HIIT to aerobic training and HIIT. |
| Rasmussen 2023 | Pain pressure thresholds decreased following exercise but not control. There was further decrease in in these thresholds after 3-month follow up for the exercise group only. |
| **Pain medication** | |
| Barbosa | Pain medication decreased in all groups. The decrease in the intervention group was not different to the decrease in the non-intervention group. |
| Cormie 2013 | No change in pain medication throughout the intervention. |
| Irwin 2015 | The number of women taking pain medication decreased rom 47% to 39% over the study period but there was no difference between groups. |
| Stigt 2013 | Use of analgesics decreased over time in both groups, but the active group had higher analgesic use than controls following 3-months of activity. |
| **Physical activity type** | |
| Ali 2021 | There was a decrease in pain following both aqua based training and land exercises. The decrease was larger for the aqua therapy group (p=0.001). |
| Barbosa 2021 | There was a decrease in pain following Pilates but not circuit training. Post intervention mean (95%CI) = 3.00(1.00, 4.75) for Pilates and 4.50(3.0, 7.0) for circuit training. |
| Courneya 2013 | There was a greater improvement in pain following high dose aerobic exercise compared to combined aerobic and resistance (mean difference = 2.0 (0.1, 3.8)) but no difference between a lower dose of aerobic and combined training (mean difference = 0.3 (-1.5,, 2.1)) |
| Garcia-Sodan 2020 | Pain improved following aerobic training but not strength training or aqua fitness. |
| Jensen 2014 | There were no clear differences between pain changes for resistance exercise (mean difference = 3.0 (-20.9, 26.9)) or aerobic exercise (mean difference = -6.6 (-38.1, 24.7)). |
| Litterini 2013 | There were non-significant decreases in pain following both cardiovascular and resistance training. Post intervention mean (95%CI) = 1.59 (-5.9, 9.07) for cardiovascular and 1.83(-5.13, 8.79) for resistance training. |
| Odynets 2018 | There were improvements in the sensory, affective, and cognitive quality of pain following water aerobics, swimming and Pilates, or Yoga and stretching exercise. Sensory changes were greatest in the Yoga group while cognitive changes were smallest in the Yoga group/ |
| OptiTrain (Bolam 2019, Mijwel 2018) | Resistance training combined with HIIT increased pain pressure thresholds at the trapezius and gluteal muscles, but aerobic training combined with HIIT did not change pain pressure thresholds. |
| Pelzer, 2023. | Development of pain was different for aerobic training and resistance training. There was an increase following aerobic training and a decrease following resistance training (p = 0.031, η2 = 0.049). |
| Schmidt 2015 | Pain improved following endurance training (p=0.07) but not following resistance training (p=0.54). |
| Vardar Yagil 2015 | Both aerobic exercise alone and aerobic exercise combined with Yoga improved pain. The mean difference (SD) was -30.70 (15.96) for the Yoga and aerobic group and -20.63 (15.77) for the aerobic only group. |
| Zengin 2017 | Pain during motion and rest improved after both Pilates and combined training. There were no clear differences between groups. |
| Vandenbyl 2017 | No clear differences in effect of strength training or Qigong on pain (p = 0.67). |
| **Physical activity dose** |  |
| Ax 2022 | Decrease in pan was greater following low to moderate intesity activity compared to high intensity activity, but the change was clinically trivial. Mean difference = 4.7, 95%CI = 0.9, 8.5. |
| Brown 2018 | Changes in pain favoured high dose compared to low dose activity but these differences were not significant. |
| Cormie 2013 | Neither heavy nor light resistance exercise significantly improved pain scores, pain severity, pain interference, or bodily pain compared to the control group. |
| Courneya 2013 | High volume aerobic exercise improved bodily pain more than a standard aerobic exercise program. Mean difference = 2.3, 95%CI = 0.5, 4.1 |
| Norris 2015 | There was no difference in bodily pain following 2- compared to 3-days per week of exercise Mean difference = 0.8, 95%CI = -5.5, 7.1 |
| Bloomquist 2019 | Low and high intensity group exercise resulted in greater decreases in pain than a walking program. Mean difference = -0.8 (-1.5, -0.1). |
| Clifford 2021 | Exercise induced hypoalgesia was more evident following high intensity exercise. |
| Cormie 2013 | Pain and pain interference were reduced 24 and 72 hours following low load resistance but not high load resistance. Although no clear differences in final values between groups. |
| Cormie 2016 | No clear differences in pain 24 hours after exercise following low-, moderate-, or high-load resistance exercise. |
| **Physical activity setting or delivery method** | |
| Bloomquist 2019 | Supervised group exercise resulted in greater decreases in pain than a home-based walking program with a pedometer. Mean difference = -0.8 (-1.5, -0.1). |
| Brocki 2014 | Bodily pain increased following supervised exercise and at home physical activity. The increase was smaller in the supervised exercise group. |
| Park 2015 | Pain decreased with oncologist recommendation of exercise, but the decrease was not greater when combined with a motivational package. |
| Steindorf 2019 | There were no differences in pain scores between supervised resistance training, home-based resistance training, and control at the end of the study period. |
| Su 2017 | Pain was not different for home based physical therapy exercises compared to outpatient physical therapy. |
| Vallerand 2018 | Post intervention pain scores were not different between telephone counselling and self-directed exercise groups. |
| Van Vulpen 2016 | There were no differences between Onco-Move (home based) and OnTrack (supervised) in pain at the end of chemotherapy (SMD = 3, 95%CI = -4.5, 10.5) or 6-months following chemotherapy (SMD = 0, 95%CI = -6.8, 6.9). |
| Zengin Alpozgen 2017 | Pain improved in supervised and home-based groups, with no clear differences between groups. |
| **Timing of physical activity** | |
| Bland 2019 | There was no difference in shooting or burning pain symptoms related it peripheral neuropathy for the exercise delivered during or after taxane chemotherapy. |
| Dodd 2010 | No difference in pain for exercise delivered during or after cancer treatment. |
| Do 2015 | The early exercise group experienced changes in pain sooner than the delayed group, but pain scores were not notably different at study conclusion. |

**Table 9. Results from individual observational studies**

| **Study** | **Finding** |
| --- | --- |
| Alfano 2007 | Post-diagnosis moderate to vigorous physical activity was not related to arm pain or breast pain. |
| Belanger 2011 | Bodily pain improved (less bodily pain) with increasing levels of physical activity (p<0.001). |
| Branstrom 2015 | Greater frequency/ reporting of physical inactivity was related to increased pain. OR (95%CI) = 3.04 (1.46, 6.32). |
| Cho 2012 | No clear difference in pain between exercisers and non-exercisers. |
| Dore 2022 | Moderate to vigorous physical activity not significantly related to pain. B (95%CI) = − 0.053 (− 0.113, 0.007) |
| Feng 2023 | Changes in daily physical activity were not associated with pain. |
| Flowers 2021 | Compared to light physical activity, moderate (B = -0.309 (-0.602, -0.015) and vigorous (B = -0.397 (-0.936, 0.142) physical activity was related to reduced neuropathic pain. |
| Klein 2021 | Physical activity was not clearly related to pain intensity (p = 0.3). |
| Sande 2014 | Change in sitting and lying or time spent standing related to change in worst and average pain. Chage in number of steps not related to changes in worst or average pain. |
| Solberg Nes 2012 | Changing physical activity related to changes in the frequency and severity of pain. More physical activity was related to better pain and symptom control. |
| Tuomi 2023 | Compared to patients with a lower pre-treatment activity, higher pre-treatment physical activity levels were related to less pain at12 months follow-up. |
| Zhuang 2023 | Physical activity not clearly related with time to deterioration in pain. HR (95%CI) = 0.91 (0.70-1.17) |
